# Development and External Validation of an Artificial Intelligence-Based Method for Scalable Chest Radiograph Diagnosis: A Multi-Country Cross-Sectional Study

**DOI:** 10.34133/research.0426

**Published:** 2024-08-06

**Authors:** Zeye Liu, Jing Xu, Chengliang Yin, Guojing Han, Yue Che, Ge Fan, Xiaofei Li, Lixin Xie, Lei Bao, Zimin Peng, Jinduo Wang, Yan Chen, Fengwen Zhang, Wenbin Ouyang, Shouzheng Wang, Junwei Guo, Yanqiu Ma, Xiangzhi Meng, Taibing Fan, Aihua Zhi, Dawaciren, Kang Yi, Tao You, Yuejin Yang, Jue Liu, Yi Shi, Yuan Huang, Xiangbin Pan

**Affiliations:** ^1^ Department of Cardiac Surgery, Peking University People’s Hospital, Peking University, Xicheng District, Beijing, China.; ^2^ State Key Laboratory of Cardiovascular Disease, Fuwai Hospital, National Center for Cardiovascular Diseases, Fuwai Hospital, Chinese Academy of Medical Sciences, and Peking Union Medical College, Beijing, China.; ^3^ Department of Structural Heart Disease, National Center for Cardiovascular Disease, China & Fuwai Hospital, Chinese Academy of Medical Sciences & Peking Union Medical College, Beijing 100037, China.; ^4^ National Health Commission Key Laboratory of Cardiovascular Regeneration Medicine, Beijing 100037, China.; ^5^ Key Laboratory of Innovative Cardiovascular Devices, Chinese Academy of Medical Sciences, Beijing 100037, China.; ^6^ National Clinical Research Center for Cardiovascular Diseases, Fuwai Hospital, Chinese Academy of Medical Sciences, Beijing 100037, China.; ^7^ Medical Big Data Research Center, Medical Innovation Research Division of Chinese PLA General Hospital, Beijing, China.; ^8^ National Engineering Research Center for Medical Big Data Application Technology, Chinese People’s Liberation Army (PLA) General Hospital, Beijing, China.; ^9^ College of Pulmonary & Critical Care Medicine, Chinese PLA General Hospital, Beijing, China.; ^10^ Center for Health Policy Research and Evaluation, Renmin University of China, Beijing, China.; ^11^ School of Public Administration and Policy, Renmin University of China, Beijing, China.; ^12^ Lightspeed & Quantum Studios, Tencent Inc., Shenzhen, China.; ^13^ Department of Cardiology, Fuwai Hospital, Chinese Academy of Medical Sciences & Peking Union Medical College, Beijing, China.; ^14^ Shenzhen Benevolence Medical Sci&Tech Co. Ltd., Shenzhen, China.; ^15^ University of Science and Technology of China, School of Cyber Science and Technology, Hefei 230000, China.; ^16^ Department of Respiratory and Critical Care Medicine, Peking Union Medical College Hospital, Chinese Academy of Medical Sciences & Peking Union Medical College, Beijing, China.; ^17^ Peking University Third Hospital, Beijing, China.; ^18^ Department of Thoracic Surgical Oncology, National Cancer Center/Cancer Hospital, Chinese Academy of Medical Sciences and Peking Union Medical College, Beijing 100021, China.; ^19^ Department of Pediatric Cardiac Surgery, Zhengzhou University Fuwai Central China Cardiovascular Hospital, Zhengzhou, Henan 450000, China.; ^20^ Fuwai Yunnan Cardiovascular Hospital, Department of Medical Imaging, Kunming 650000, China.; ^21^ The Autonomous Region People’s Hospital, Xizang, China.; ^22^ Department of Cardiovascular Surgery, Gansu Provincial Hospital, Lanzhou, China.; ^23^ Gansu International Scientific and Technological Cooperation Base of Diagnosis and Treatment of Congenital Heart Disease, Lanzhou, China.; ^24^ Department of Epidemiology and Biostatistics, School of Public Health, Peking University, Beijing, China.; ^25^ State Key Laboratory of Cardiovascular Disease, Fuwai Hospital, National Center for Cardiovascular Diseases, Cardiac Surgery Center, Fuwai Hospital, Chinese Academy of Medical Sciences, and Peking Union Medical College, Beijing, China.

## Abstract

**Problem:** Chest radiography is a crucial tool for diagnosing thoracic disorders, but interpretation errors and a lack of qualified practitioners can cause delays in treatment. **Aim:** This study aimed to develop a reliable multi-classification artificial intelligence (AI) tool to improve the accuracy and efficiency of chest radiograph diagnosis. **Methods:** We developed a convolutional neural network (CNN) capable of distinguishing among 26 thoracic diagnoses. The model was trained and externally validated using 795,055 chest radiographs from 13 datasets across 4 countries. **Results:** The CNN model achieved an average area under the curve (AUC) of 0.961 across all 26 diagnoses in the testing set. COVID-19 detection achieved perfect accuracy (AUC 1.000, [95% confidence interval {CI}, 1.000 to 1.000]), while effusion or pleural effusion detection showed the lowest accuracy (AUC 0.8453, [95% CI, 0.8417 to 0.8489]). In external validation, the model demonstrated strong reproducibility and generalizability within the local dataset, achieving an AUC of 0.9634 for lung opacity detection (95% CI, 0.9423 to 0.9702). The CNN outperformed both radiologists and nonradiological physicians, particularly in trans-device image recognition. Even for diseases not specifically trained on, such as aortic dissection, the AI model showed considerable scalability and enhanced diagnostic accuracy for physicians of varying experience levels (all *P* < 0.05). Additionally, our model exhibited no gender bias (*P* > 0.05). **Conclusion:** The developed AI algorithm, now available as professional web-based software, substantively improves chest radiograph interpretation. This research advances medical imaging and offers substantial diagnostic support in clinical settings.

## Introduction

Chest radiography stands as one of the most frequently performed imaging procedures worldwide for evaluating lung parenchyma, airways, heart, and mediastinum disorders, owing to its portability, widespread availability, and affordability [1]. The significance of diagnostic chest radiographs is undeniable, with their usage skyrocketing, notably in the face of the ongoing coronavirus disease 2019 (COVID-19) pandemic. However, several challenges can mar the diagnostic accuracy of chest radiographs. First, interpreting these images is a complex endeavor that necessitates radiologists to possess sophisticated practical skills and analytical acumen to guarantee precise diagnostic outcomes. Despite their expertise, interpretation errors are an unfortunate inevitability, potentially leading to misdiagnosis and subsequent delays in patient treatment [2,3]. Second, the scarcity of qualified radiologists, which is not keeping pace with the escalating number of examinations, amplifies the risk of diagnostic errors. Consequently, the burden of chest radiography is on the rise in both developed and developing nations, with reporting delays emerging as an increasingly pressing concern [3–5]. To address these issues, our proposed approach leverages a cutting-edge convolutional neural network (CNN), trained on the most extensive dataset to date, aiming to introduce a robust multi-category artificial intelligence (AI) tool. This AI-driven solution is poised to enhance diagnostic precision, alleviate the workload on radiologists, and ultimately expedite patient care.

Recent years have witnessed a surge in exploring the development and implementation of AI within the medical domain, spanning various clinical areas such as oncology [6], cardiovascular diseases [7–9], and autoimmune diseases [10]. Across these domains, AI models have demonstrated expert-level diagnostic accuracy. Moreover, there is an increasing emphasis on utilizing machine learning (ML) techniques in medical imaging applications, attracting significant attention from clinical, research, and socioeconomic perspectives [11]. On the one hand, the perceptual and cognitive capabilities of AI can be used to identify and extract crucial information from medical images, thereby enhancing the efficiency of interpreting radiographs [12]. Conversely, ML enables the integration of extensive imaging data and clinical information, empowering AI models to concurrently undertake diagnostic tasks, thereby potentially reducing radiologist misdiagnosis rates [13]. Recent studies have underscored the significant performance enhancements achievable by ML models when trained on large datasets. Notably, advancements such as the generative pre-trained transformer and segment anything models have demonstrated remarkable improvements in this regard [14,15]. However, current research on AI in chest radiography grapples with several limitations, including restricted classification categories for abnormalities, biases stemming from insufficient sample sizes, limited generalizability across diverse populations, and challenges in recognizing images obtained from different devices [16–18]. Furthermore, current research on using AI methods to assist in disease diagnosis still faces various limitations. For instance, due to constraints in sample collection capabilities, the sample sizes are often small [19,20], with some studies involving only a few dozen cases, which limits the reliability and generalizability of the models. Additionally, the number of detectable disease labels in existing models is very limited [21], restricting the application of this technology in clinical practice. Some studies have also made modifications to the models to reduce training costs and time, but this has somewhat compromised the reliability of validation [22].

In this study, these challenges were addressed by developing a CNN tailored to discern 26 thoracic diagnoses. Data from 13 datasets spanning 4 countries, totaling 795,055 chest radiographs, were incorporated. Our study methodology, rooted in ResNet 50, incorporates an innovative approach where each disease is modeled with an independent binomial distribution. This framework enables efficient training on large-scale chest radiograph datasets encompassing multiple diagnoses. Furthermore, to enhance the robustness of our model, chest radiographs obtained from mobile phones sourced from open-access databases, along with data from local hospitals, were incorporated. This integration facilitated fine-tuning of our model, resulting in superior stability and competitiveness in complex environments. Moreover, our AI algorithm was transformed into professional web-based software. This adaptation allows for potential deployment on mobile servers, facilitating user-friendly access for both the general population and medical professionals. This advancement holds promise for streamlining the chest radiography diagnosis and treatment process in the future.

### Contributions list

**•** Model: Developed a novel model for chest radiograph detection, utilizing independent distribution modeling to enhance the efficiency and stability of the CNN model. This approach significantly improves model performance.

**•** Application: Designed a framework accessible via mobile devices, including a demo to facilitate initial detection by nonprofessional users. This framework can effectively serve large-scale user access.

**•** Experiments: Conducted experiments on 13 datasets, achieving an average area under the curve (AUC) of 0.961 across all 26 thoracic diagnoses in the testing set. In external validation, the model demonstrated excellent reproducibility and generalizability, achieving an AUC of 0.9634 for lung opacity detection [95% confidence interval (CI), 0.9423 to 0.9702].

**•** Performance: The model outperformed both radiologists and nonradiologists, especially in scenarios requiring cross-device image recognition, highlighting its superior proficiency.

**•** Scalability: Demonstrated significant scalability and potential to enhance the accuracy of chest radiograph interpretation among physicians with varying levels of experience and expertise (all *P* < 0.05).

**•** Bias analysis: Analysis revealed no evidence of gender bias in the model’s predictions (*P* > 0.05).

## Results

### Internal and external diagnostic performance of the CNN model

**Fig. 1.**
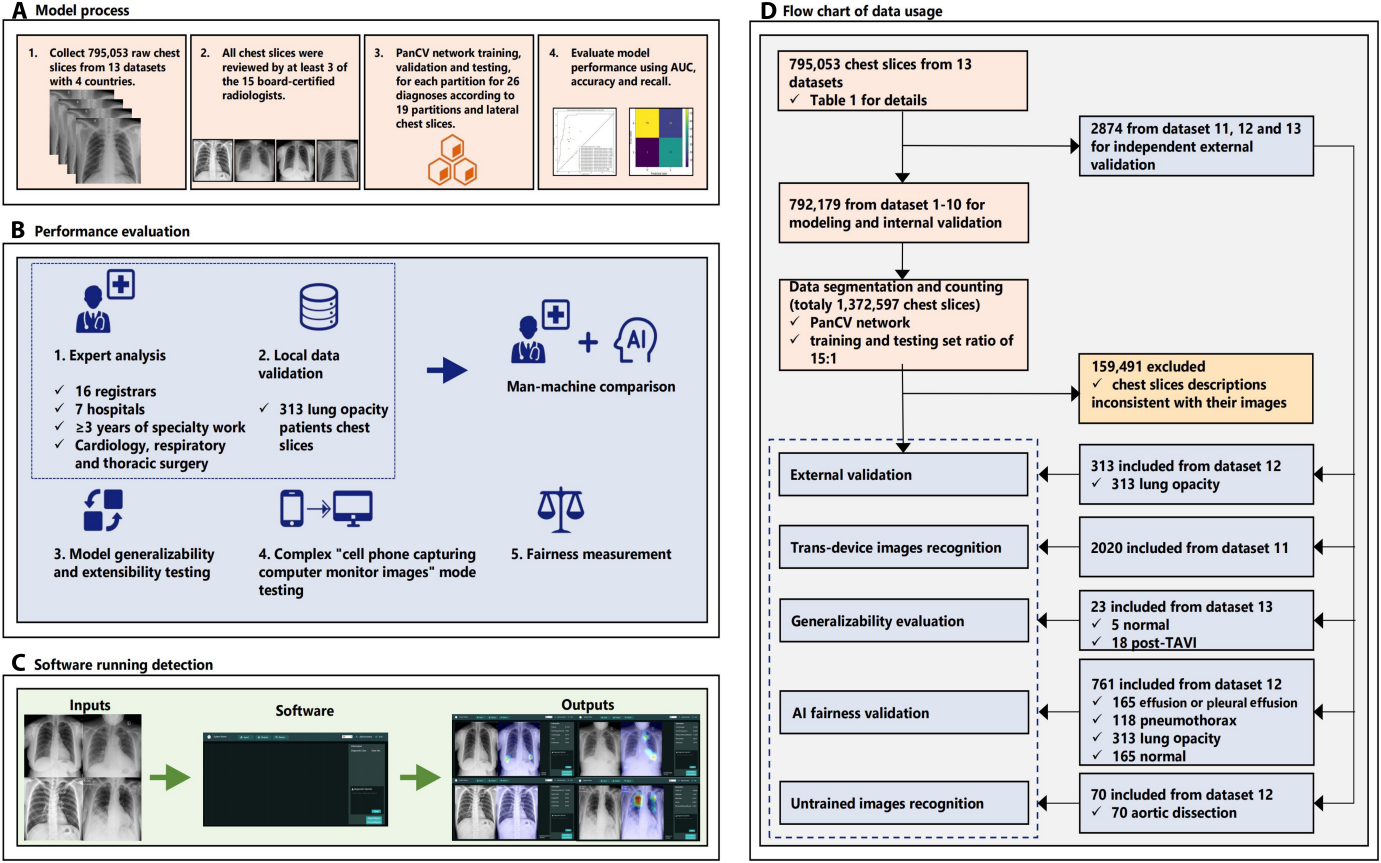
(A to D) Research flowchart for the development and validation of PanCV software. AUC, area under the receiver operating characteristic curve; TAVI, transcatheter aortic valve implantation; COVID-19, coronavirus disease 2019.

**Fig. 2.**
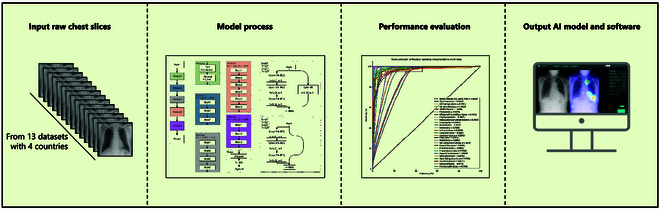
PanCV model architecture. The PanCV model architecture is composed of multiple convolutional layers and a fully connected layer. The input image is first processed by the convolutional layers, which extract features from the image. The output of the convolutional layers is then flattened and passed through the fully connected layer, which produces the final classification output. The PanCV model architecture has shown high accuracy in classifying images in various applications.

**Fig. 3.**
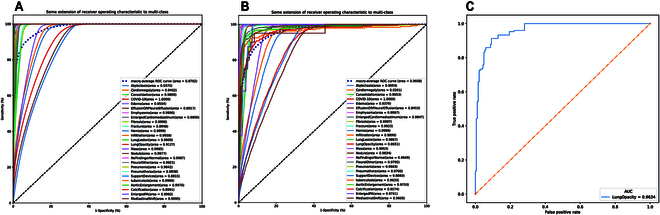
Results of PanCV model training (A), test (B), and local validation (C). The local validation data were obtained from 301 hospitals.

The research process is shown in Figs. 1 and 2. The AUCs of the algorithm for each diagnosis in both the training and testing sets are presented in Fig. 3A and B, respectively. In the testing set, the CNN model achieved an average AUC of 0.961 for all 26 diagnoses. Notably, COVID-19 detection yielded the highest AUC, with the model achieving perfect accuracy (1.000, [95% CI, 1.000 to 1.000]). Conversely, detection of Effusion or pleural effusion demonstrated the lowest diagnostic accuracy (AUC, 0.8453, [95% CI, 0.8417 to 0.8489]).

In the external validation, the CNN model showed excellent reproducibility and generalizability within the local dataset, boasting an AUC of 0.9634 for lung opacity detection (95% CI, 0.9423 to 0.9702). Remarkably, this outperformed the AUC obtained for the in-house testing set (Fig. 3C), indicating the stability of our algorithm in identifying previously unseen chest radiographs.

The proposed method integrates 2 essential components: the convolutional block and the identity block. The convolutional block introduces nonlinear transformations, whereas the identity block preserves the dimensionality of the input features and learns an identity mapping. Both blocks consist of a sequence of convolutional layers, batch normalization layers, and activation functions. The kernel size, filters, and strides are critical parameters that define these blocks. The specific settings of each block are detailed in Table S3. Additionally, Table S4 provides the details of the hyperparameters used in model training. For a comprehensive evaluation of the proposed model, Table S5 presents the PanCV’s performance metrics on the test set, including the F1 score, specificity, recall score, and top-*K* error rate.

### Cognitive ability and trans-device image recognition

**Fig. 4.**
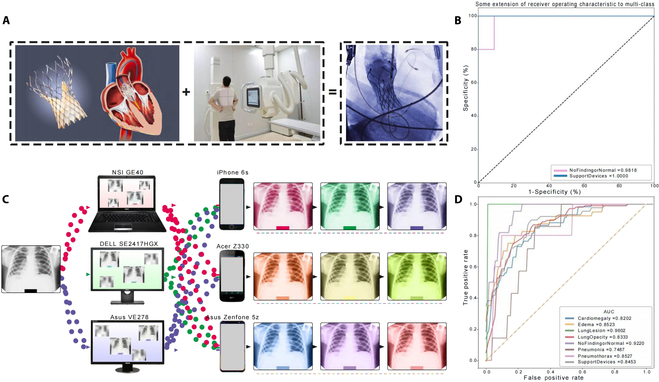
Cognitive ability and trans-device image recognition of the PanCV model. (A) Postoperative chest radiograph of a TAVI patient obtained from the Fuwai Yunnan Cardiovascular Hospital. (B) Validation of the model on postoperative chest radiographs of TAVI patients and normal chest radiographs obtained from the same hospital. (C) Schematic representation of the complex environment dataset, which included 3 types of displays and 3 types of camera phones. (D) Model performance on the complex environment dataset.

To further validate the reliability and extension performance of our AI model, we assessed chest radiographs from post-TAVI (transcatheter aortic valve implantation) patients and normal chest radiographs that were not part of the training dataset. Metallic stents in the heart, implanted during TAVI surgery, may appear in chest radiographs, although they might not always be clearly visible and can be overlooked by less experienced healthcare workers. Despite the absence of similar chest radiographs in our training data, the distinct appearance of TAVI valve stents from human anatomical structures enables AI to accurately classify them through extrapolation. Our results demonstrated the model’s accurate identification of both supportive devices (AUC, 1.000, [95% CI, 1.000 to 1.000]) and normal chest radiographs (AUC, 0.9818, [95% CI, 0.9200 to 1.000]; Fig. 4A and B).

Applying AI in complex clinical environments poses a significant challenge in the field. We tested the performance of our AI model in real-world clinical settings, including chest radiographs captured with mobile phones and under different lighting conditions. This process mimics the clinical practice of seeking assistance from radiology department colleagues for diagnosis, demanding more from AI models. Our analysis revealed the model’s highest AUC of 0.9602 (95% CI, 0.8907 to 1.0000) for identifying lung lesions and the lowest AUC of 0.7487 (95% CI, 0.6023 to 0.8951) for identifying pneumonia, demonstrating the model’s good performance in trans-device image recognition (Fig. 4C and D). Furthermore, the analysis in this section was conducted using external datasets, which further demonstrates our model’s superior generalizability.

### Comparison of the preliminary read performance of the CNN model versus clinical physicians

**Fig. 5.**
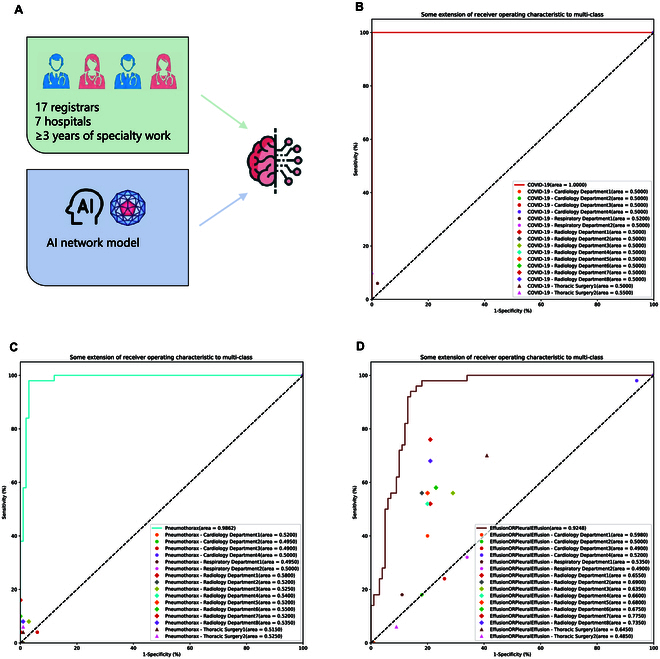
Comparison of the preliminary read performance of the CNN model versus clinical physicians. (A) Seventeen physicians, including cardiology, respiratory, and thoracic surgeons, registered at 7 hospitals each with more than 3 years of specialty work were compared with AI. The 3 categories with the best, worst, and median (AUC) machine performance on the validation set—COVID-19, pneumothorax, and effusion or pleural effusion—were selected for human–machine comparison testing. (B) Results of COVID-19 interpretation comparison. (C) Results of pneumothorax interpretation comparison. (D) Results of effusion or pleural effusion interpretation comparison.

Based on the testing results of our previous model, we selected COVID-19 (Fig. 5B), pneumothorax (Fig. 5C), and effusion or pleural effusion (Fig. 5D) for a comparative study on the accuracy of human–machine reading according to the AUC values. Our findings indicated that our model outperformed both radiologists and nonradiological physicians. Specifically, for COVID-19, the model achieved an AUC of 1.000, whereas the highest AUC achieved by physicians was 0.5500. Similarly, for pneumothorax, the model yielded an AUC of 0.9862, compared to the highest physician AUC of 0.5800. Even in the case of effusion or pleural effusion, where the CNN model exhibited the lowest performance, it still markedly surpassed professional physicians in terms of diagnostic accuracy (AUCs ranged from 0.4850 to 0.7750 for the physicians, AUC of 0.9248 for the CNN model). Furthermore, the physician group consisted of experts from diverse medical fields, including radiology, suggesting that AI models can offer benefits to physicians across various medical specialties.

### Application of CNN-based model in untrained diseases and its optimization of medical process

**Fig. 6.**
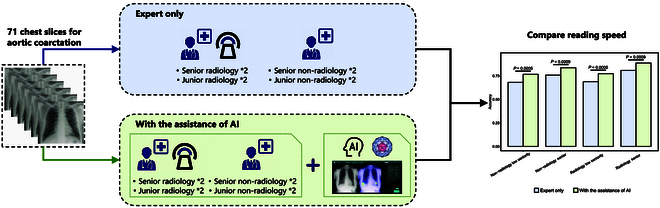
Results of the paired study of chest radiographs for aortic dissection. The research workflow diagram and the improvement in reading accuracy for 4 groups of physicians (i.e., low-experience nonradiologists, high-experience nonradiologists, low-experience radiologists, and high-experience radiologists) by AI are shown. The consistency of different groups was evaluated here using paired McNemar’s test.

As shown in Fig. 6, notable intergroup disparities in standalone chest radiograph interpretation accuracy were observed among the 4 expert groups. Our data showed accuracy rates of 0.680851 for low-experience nonradiologists, 0.757143 for high-experience nonradiologists, 0.687943 for low-experience radiologists, and 0.807143 for high-experience radiologists. Within the radiologist cohort, low-experience experts exhibited lower accuracy compared to their high-experience counterparts (*P* = 0.0061). Compared with standalone performance, the assistance of AI can narrow the gap in the level of accuracy between radiologists and nonradiologists (Fig. 6). The accuracy rates for low-experience nonradiologists, high-experience nonradiologists, low-experience radiologists, and high-experience radiologists increased to 0.765957, 0.835714, 0.77305, and 0.885714, respectively. All groups demonstrated significant accuracy improvements with AI assistance (all *P* < 0.05; Fig. 6).

Furthermore, our results suggest that AI assistance enhances the accuracy of low-experience and nonradiologist experts. Notably, low-experience nonradiologists may be able to achieve a higher level of accuracy in medical image interpretation than high-experience nonradiologists and low-experience radiologists when aided by AI algorithms. This improvement in accuracy holds paramount importance for expediting diagnosis and minimizing missed diagnoses of critical conditions such as aortic dissection. Although “aortic dissection” is not one of the 26 labels, experimental evidence shows that diagnoses suggested by AI models, such as “Effusion OR Pleural Effusion” and “Enlarged Cardiomediastinum,” can effectively help physicians draw the conclusion that “this chest radiograph is abnormal and needs further examination.”

### Fairness analysis of different gender populations

**Fig. 7.**
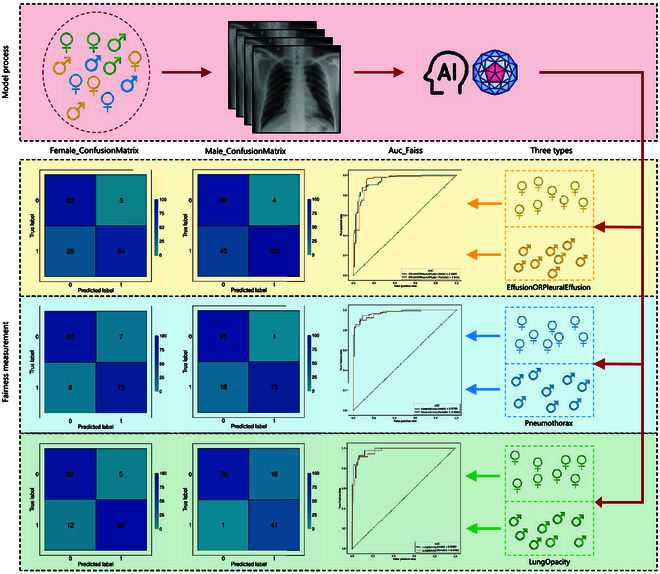
Fairness analysis of the PanCV model. Chest radiographs with diagnoses of “Effusion OR Pleural Effusion,” “Pneumothorax,” and “Lung Opacity” were used to verify the fairness of the AI model; specifically, we tested whether the model’s classification differed between male and female chest radiographs. The above 3 labels represent the 3 categories of the model's worst, second-worst, and median (AUC) performance on the validation set.

We conducted a fairness analysis of our model across different gender groups, calculating the AUCs of effusion or pleural effusion, pneumothorax, and lung opacity detection separately for male and female patients. Our finding revealed consistent model performance across both genders (Fig. 7). Furthermore, statistical analysis of the model’s performance indicated no significant difference between male and female patients (Fig. 7). These data underscored the absence of gender bias in our model.

### CNN model-aided diagnostic software application

**Fig. 8.**
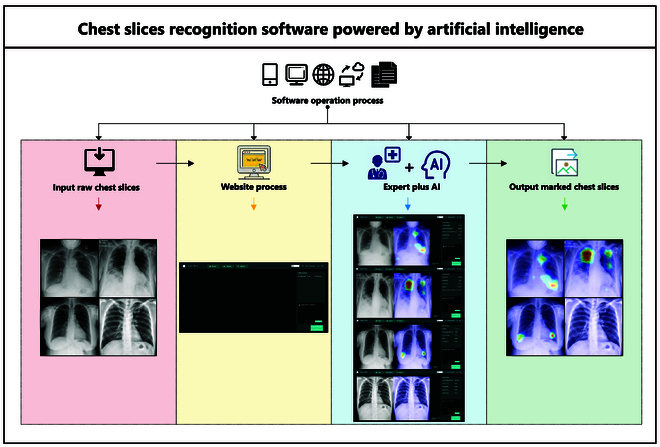
Demonstration of software usage and displayed results.

To disseminate our model with those who could benefit from it, we seamlessly integrated it into a robust real-world system and developed a user-friendly application accessible at https://tfpic.benefm.com/. Due to cost considerations, the current product demo employs an invitation code registration system. Interested parties are encouraged to contact the corresponding author to request an invitation code. Our versatile application caters not only to the preliminary screening of nonspecialist experts but also to inquiries from potential patients in the comfort of their homes. The user-friendly interface of the front-end page, depicted in Fig. 8, facilitates ease of use, while the back-end system, as illustrated in the same figure, comprises 2 essential modules: the model server module and the logic module. To demonstrate the powerful functionality of the software, we randomly selected a subset of chest radiographs across the 26 diagnostic labels and input them into the software for interpretation and heatmap graphics generation, as shown in Figs. S8 to S16. This also serves as a demonstration of the interpretability of our AI model.

One of the standout features of our proposed framework is its remarkable scalability. By simply scaling up the number of servers, our system can gracefully accommodate a significant influx of users. The predictive pipeline is purposefully designed to be independent, thereby ensuring seamless expansion and enabling efficient handling of substantial user volumes. Consequently, our system adeptly serves an expanding user base without compromising performance or responsiveness.

## Discussion

In this study, we developed a CNN-based model for chest radiograph diagnosis. In the PanCV model, we have opted to utilize independent sigmoid functions instead of the softmax activation function for modeling the output probabilities of various diseases. While this adjustment may appear straightforward, we contend that it represents a significant improvement for amalgamating high-level information from disparate disease labels (Table S6). Compared to currently published studies (Table S7), our study includes larger and more diverse datasets, incorporating 795,055 chest radiographs using multicenter data from China, the United States, Brazil, and Vietnam for modeling, and validated using 3 independent datasets from different countries. Moreover, our algorithm exhibits significant advancements in multicategorical detection, capable of identifying 26 common thoracic diagnoses in both the testing and external validation sets. This model serves as a comprehensive diagnostic tool for medical professionals [23]. The consistent performance of the multiclass algorithm across diverse populations suggests its generalizability and reliability, catering to individuals with varied demographics, cultural backgrounds, and health conditions [18,24].

Impressively, our model significantly outperformed radiologists, cardiologists, pulmonologists, and thoracic surgeons in diagnostic accuracy for the 3 selected diseases, underscoring its potential to support clinical decision-making. During the COVID-19 pandemic, healthcare systems all over the world have been overwhelmed and faced critical shortages of equipment and supplies [25]. As curative treatments for COVID-19 were not yet available, early identification and supportive care for infected individuals became a crucial strategy in controlling virus transmission. While nucleic acid testing via reverse transcription polymerase chain reaction (RT-PCR) remains the primary method of COVID-19 diagnosis, attempts have been made to develop AI-based diagnostic models [26–29]. Importantly, our AI model achieved a perfect AUC of 1.000 in both the training and testing sets for identifying COVID-19, demonstrating superior diagnostic accuracy compared to expert physicians in the human–machine comparative analysis. Although these models have not yet been established as diagnostic standards for COVID-19, they may prove useful to clinicians when integrated with clinical signs and symptoms [30].

Notably, one of the key innovations of our model is the segmentation of chest radiographs into 20 individual partitions for analysis, which integrates anatomical structures with clinical practices, enabling radiologists to promptly locate lesions and potential areas of concern within the image. The 20-partition algorithm, coupled with gradient-weighted class activation mapping (Grad-CAM), significantly enhances the explainability of our model via class-discriminative visualization, pinpointing structures within the fields and areas surrounding the suspected lesions in each chest radiograph [31,32]. By leveraging common disease locations and AI-generated suggestions, radiologists are able to further scrutinize these targeted sections and confirm their final diagnosis more efficiently [23], facilitating prompt initiation of necessary treatments and improving patient outcomes. Taking the 3 categories with the best, worst, and median (AUC) model performance on the validation set, namely, COVID-19, pneumothorax, and effusion or pleural effusion (i.e., the 3 labels used for human–machine comparison) as examples. COVID-19 often presents as bilateral lesions on chest radiographs, predominantly in the peripheral regions of the lungs [33]. This observation aligns with the findings depicted in Fig. S9, while Figs. S1 to S7 indicate consistent diagnostic performance across the 19 partitions, except for lateral chest radiographs. This suggests that COVID-19 does not exhibit an absolute site of predilection, consistent with current clinical experience [33,34]. Pneumothorax typically manifests as loss of the visceral pleural line and peripheral lung markings, possibly accompanied by small amounts of pleural fluid [35]. This is corroborated by the Grad-CAM results in Fig. S15, and by the relatively higher AUC in the 2 outer bands (partitions 1 to 3 and 16 to 18) in Figs. S1 to S7. Effusion or pleural effusion typically presents as blunting of the costophrenic angle and homogeneous increased density [36]. The areas of costophrenic angle and increased density are consistent with the key areas marked by Grad-CAM in Fig. S10. Other findings, such as “Nodule,” are also marked clearly at the site of the nodules, while normal chest radiographs remain marked (Fig. S14). Therefore, the utilization of the 20-partition algorithm combined with Grad-CAM effectively enhances diagnostic efficiency and accuracy for clinical physicians.

In addition to heightened efficiency, the AI model has the potential to enhance diagnostic accuracy. Our analyses indicated significant enhancements in diagnostic accuracy for junior and nonradiological specialist physicians with the use of AI. This finding holds particular significance in emergency and critical care settings. Furthermore, we conducted an analysis of the fairness of the CNN model across different gender populations, with results suggesting fairness and impartiality with regard to gender, enabling equal application to male and female patients. However, we acknowledge the possibility of other factors contributing to bias in medical AI models, emphasizing the necessity for further research to comprehensively understand and address these issues.

While imaging research has experienced a proliferation of literature on novel diagnostic, classification, and prediction tools developed using ML techniques in recent years, there remains a paucity of studies evaluating the practicality and real-world applicability of these models in clinical practice [37]. A notable strength of our AI model lies in its ability to generalize and recognize previously untrained chest radiographs, such as those obtained after TAVI procedure, thereby highlighting its adaptability for continuous learning, refinement, and potential for broader application in clinical settings. Additionally, the use of AI for trans-device image recognition has been a challenge due to various factors, including camera resolution, image distortions, and shooting distance [38]. Nevertheless, our model has shown robust diagnostic performance even when analyzing chest radiographs captured by smartphone cameras of computer screens, demonstrating its utility in complex and real-world scenarios. The integration of 5G technology and AI holds promise for revolutionizing remote medical imaging [39]. In remote diagnosis and grading, the AI model can furnish a preliminary assessment, subject to further review and confirmation by a human expert [40]. This approach has the potential to alleviate the workload of healthcare professionals and improve the efficiency of diagnosis, particularly in regions with limited medical resources. The accurate diagnosis of images captured through mobile devices also enables access to remote consultations and telemedicine, overcoming geographical barriers and extending medical services to more people in need, potentially reshaping the healthcare landscape in the years ahead. Moreover, this method can also reduce the contact between patients and doctors, decreasing the risk of cross-infection and providing robust medical security during an epidemic [41,42]. To fully realize the potential of our model, we have developed a convenient and user-friendly software for its implementation, easily accessible and operable by healthcare professionals and patients alike. This represents a significant stride forward in medical imaging, with profound potential impacts on patient outcomes and healthcare service delivery.

From a health economics perspective, developing countries like China, Brazil, and Vietnam face challenges with inadequate levels of medical services and health human resources. Similarly, in developed countries such as the United States, there exist significant developmental disparities among different regions. Our model has the potential to substantially mitigate the imbalance of regional health resources and service capabilities, thereby fostering health equity across various countries and regions worldwide.

Several limitations apply to our study. First, while a larger sample size and more diverse data from multiple centers could potentially yield even better results, it is noteworthy that our CNN-based model is already trained on a relatively large sample size among those reported in published literature. Second, our study is constrained by the inclusion of a limited set of diagnostic labels. Nevertheless, the 26 labels encompass a wide range of diseases in chest radiograph diagnosis and can offer indications for abnormal phenomena associated with other conditions. Additionally, the labels used in our study were a combination and integration of multiple public database labels, which may lead to some overlap where multiple chest radiographs from the same patient are included in both the training and testing sets. However, given our focus on image-level tasks, it is natural to reuse multiple images from a single patient. If the task were patient-level, combining multiple predicted results from various images with multiple instances would be necessary. Hence, we adopted a data processing method that allows the reuse of multiple images from a single patient according to our research requirements [43–45]. To optimize this issue, we introduced data from Chinese People’s Liberation Army (PLA) General Hospital and Fuwai Hospital as completely independent external validation sets, devoid of such cross-over situation.

The clinical significance of our study lies in the potential for AI-assisted chest radiograph diagnosis to enhance diagnostic accuracy, reduce misdiagnoses, and ultimately improve patient care. This may lead to more timely and appropriate treatments, potentially improving patient outcomes and reducing healthcare costs. While there remain ethical and legal challenges associated with the application of AI technology in chest radiography diagnosis, we firmly believe that with ongoing technological advancements, AI-assisted chest radiography diagnosis will play an increasingly indispensable role in remote medical care. It is important to note that AI technology serves as an auxiliary diagnostic method and not a complete replacement for manual diagnosis. Therefore, while embracing AI technology, it is imperative to recognize the indispensable role of physicians in providing superior medical services to patients.

In summary, we have developed an AI-based multilabel, explainable, and highly accurate chest radiograph diagnostic model. The model showcased outstanding performance across various metrics. In the test set, it achieved an AUC of 0.961 for all 26 thoracic diagnoses. During external validation, the model demonstrated remarkable reproducibility and generalizability, attaining an AUC of 0.9634 (95% CI, 0.9423 to 0.9702) in detecting lung opacity. Moreover, the model outperformed both radiologists and nonradiological physicians, especially in scenarios requiring trans-device image recognition, highlighting its exceptional proficiency. Additionally, the model exhibited notable scalability and the potential to enhance the precision of chest radiograph interpretations among physicians with varying experience levels and expertise (all *P* < 0.05). Furthermore, our analysis revealed no evidence of gender bias in the model’s predictions (*P* > 0.05). Overall, our research represents a significant advancement in the field of medical image analysis. As AI continues to evolve as a research focus, we hope that our work will offer valuable insights and opportunities for healthcare professionals and patients.

## Conclusion

In many regions, the growth rate of healthcare professionals lags behind the increase in the number of examinations, leading to an increased workload for specialists in resource-scarce areas and delays in reporting chest radiograph interpretations. Current research on AI in chest radiograph also faces limitations, including limited classification categories, sample size biases, lack of cross-population generalizability, and inability to recognize images obtained from other devices.

To address these issues, we have developed the world’s largest chest radiograph interpretation model. Using multicenter data from China, the United States, Brazil, and Vietnam, we included 795,055 chest radiographs capable of distinguishing 26 different diagnoses. One significant advantage of our model is its ability to predict and identify chest radiographs not previously included in training, such as those taken after TAVI procedures. This highlights its continuous learning capability and enhanced adaptability, as well as its potential for broader application in clinical settings. The model demonstrated robust diagnostic performance across different devices, even when analyzing chest radiographs captured by smartphone cameras of computer screens, proving its practicality in complex real-world scenarios.

AI usage significantly improved diagnostic accuracy for junior and nonradiological specialist physicians, a finding particularly valuable in emergency and critical care settings. Our model showed fairness and impartiality regarding gender, enabling equal application to male and female patients. We developed a convenient and user-friendly software to facilitate the use of our model, representing a significant advancement in medical imaging with profound potential impacts on patient outcomes and healthcare service delivery.

Using larger sample sizes and more data from different centers to construct AI models could theoretically yield better results. The labels used in this study were a combination and integration of multiple public database labels, which might lead to the inclusion of multiple chest radiographs from the same patient in both the training and testing sets. To address potential concerns, we introduced data from local datasets as completely independent external validation sets, devoid of such cross-over situations.

Future research will explore multimodal approaches, integrating other data types for comprehensive diagnostics. Additionally, we plan to investigate the application of federated learning to enhance model performance while protecting user privacy. Continuous improvement and real-world application of the model will also be a focus, ensuring consistent updates and adaptability to maintain high diagnostic accuracy across diverse patient populations.

## Materials and Methods

### Data sources and management

For our model development and validation, a comprehensive dataset comprising 795,055 chest radiographs sourced from 13 datasets spanning 4 countries was harnessed. All datasets used in this study were collated with approval from the respective ethics committees, of which 11 were open-source datasets [46–55]. The remaining 2 local datasets were assembled upon obtaining approval from the Ethics Committee of the Fuwai Yunnan Cardiovascular Hospital and the Ethics Committee of the Chinese PLA General Hospital. Informed consent from patients was waived for these datasets. The local data encompass records from January to March 2022. Our model targets the discrimination of common and clinically significant thoracic diagnoses identifiable on chest radiographs. The 26 diagnoses encompassed in this study are atelectasis, cardiomegaly, consolidation, COVID-19, oedema, effusion or pleural effusion, emphysema, enlarged cardiomediastinum, fibrosis, fracture, hernia, infiltration, lung lesion, lung opacity, mass, nodule, no finding or normal, pleural other, pneumonia, pneumothorax, support devices, tuberculosis, aortic enlargement, calcification, enlarged pulmonary artery, and mediastinal shift [56]. Each chest radiograph in our study might represent multiple diagnoses, resulting in potential duplication when categorized by diagnosis. This variability underscores the complexity of the dataset. Moreover, the inclusion of data from diverse countries and regions introduces variability in chest radiograph quality. To further enhance the robustness of the model, the inclusion of the data was maximized in the training set while ensuring an adequate number of chest radiographs for testing. This was achieved by randomly dividing the 10 open-source datasets (excluding Photo-DEV from the 11 aforementioned datasets) into training and testing set at a 15:1 ratio. Notably, the testing set was reserved solely for performance evaluation and was not employed in model training. Consequently, the training set comprised 1,286,810 chest radiographs, while the validation set comprised 85,787 chest radiographs. After expert quality control, a total of 1,139,328 chest radiographs were retained in the training set, alongside 73,778 chest radiographs in the validation set. Further details regarding quality control are provided in the subsequent paragraph. The comprehensive data source information is presented in Table 1, while Table S1 contains patient data from PLA General Hospital and Fuwai Hospital. Table S2 displays the distribution of chest radiographs across different labels after reorganizing the 10 datasets used for modeling. Notably, many chest radiographs in these datasets were associated with multiple labels, leading to a considerable increase in the number of chest radiographs in the reorganized datasets compared to the original ones. It is pertinent to highlight that our study incorporates 13 datasets. Among these, 10 datasets were divided into a 15:1 ratio for model construction and internal validation, while the remaining 3 datasets were used for external validation (refer to Table 1 and Fig. 1 for further details). This meticulous approach was adopted to ensure the reliability of our model. Furthermore, the utilization of diverse publicly available datasets from different countries inevitably introduces inconsistencies in the definition of identical diagnoses across different datasets. This aspect underscores the need for careful consideration and validation when interpreting results. For instance, there exists ambiguity regarding the classification of a chest radiograph as a “Nodule” or a “Mass.” Additionally, challenges arise from fuzzy boundaries between different labels, such as “Lung Lesion” and “Lung Opacity,” within the MIMIC-CXR dataset [43]. Furthermore, it is possible that a single disease might manifest with multiple chest radiographic features, such as “Pneumonia” and “Pleural Effusion” [57]. Nevertheless, it is believed that these limitations do not affect the significance of our research. Primarily, our research objective is to develop a practical clinical diagnostic tool, necessitating the inclusion of extensive datasets. These datasets should encompass diverse diagnostic criteria or practices as possible to enhance the robustness of the model. Our goal is to assist rather than replace doctors. Whether the prompt is “Nodule” or “Mass,” it serves to draw the attention of physicians, expediting diagnosis. Similarly, whether AI indicates “Pneumonia” or “Pleural Effusion,” it aids in identifying lung infections or inflammatory lesions. Our research addresses the urgent clinical need for rapid screening, timely identification of at-risk populations, and prevention of missed diagnoses. Moreover, this method of data usage is widely accepted and adopted in numerous advanced research endeavors [44,55,58–60].

**Table 1.** Detailed data source information

Open-source datasets	Sample size	Reference	Serial number
MIMIC-CXR-JPG	377,110	[48]	1
CheXpert	224,316	[49]	2
NIH Chest X-ray Dataset	112,120	[50]	3
Montgomery County - Chest X-ray Database	130	https://lhncbc.nlm.nih.gov/publication/pub9931	4
Shenzhen Hospital CXR Set	662	https://lhncbc.nlm.nih.gov/publication/pub9931	5
COVID-19 RADIOGRAPHY DATABASE	3,886	[51]	6
Chest X-Ray Images (Pneumonia)	5,863	[52]	7
BRAX	40,967	[46,53]	8
VinDr-CXR	18,000	[54]	9
VinDr-PCXR	9,125	[55]	10
Photo-DEV	2,020	[47]	11
PLA General Hospital Database	831	Local validation sets	12
Fuwai Hospital Database	25	Local validation sets	13

CXR, chest x-ray; PCXR, pediatric chest x-ray; PLA, Chinese People’s Liberation Army

For data curation, all chest radiographs were evaluated by a minimum of 3 of the 15 board-certified radiologists, each with over 3 years of experience in chest radiograph interpretation involved in the study. A total of 159,491 chest radiographs, whose descriptions did not correspond to the respective images, were excluded based on the assessment of at least one radiologist. Specifically, when a chest radiograph exhibited multiple diagnoses, each diagnosis was evaluated sequentially. For instance, if a chest radiograph in the dataset contained 2 diagnoses, “Fibrosis” and “Nodule,” it was categorized under both “Fibrosis” and “Nodule.” If 3 experts concurred that the “Fibrosis” diagnosis was valid, the chest radiograph was retained under this label. Conversely, if any of the experts deemed the “Nodule” diagnosis invalid, the chest radiograph was excluded from the “Nodule” label. During this process, the experts did not modify or assign any labels to a chest radiograph.

This report conforms to the Standards for Reporting of Diagnostic Accuracy (STARD) 2015 reporting guidelines [61]. The flowchart outlining the study’s methodology is illustrated in Fig. 1.

### Development of the CNN diagnostic algorithm

Due to the unique demands of multiclassification tasks, a CNN model was designed specifically for this type of data based on the ResNet architecture and termed the PanCV network [62]. Notably, we replaced the softmax function in the original model with independent sigmoid functions. This adjustment enables the model to handle multilabel classification more effectively by computing prediction probabilities for each label independently, rather than treating them as mutually exclusive. It also enhances model robustness of the model by reducing the impact of label correlation and noise. The model-based software provides end-to-end output of predicted probabilities for 26 labels, concurrently delivering the top 5 most probable predictions to facilitate decision-making. Following training, the model comprises a total of 23,800,730 parameters. Detailed information regarding the development of the CNN model is provided in Fig. 2 and the Supplementary Materials.

To tailor our model for this specific clinical context, we incorporated a lung partitioning method commonly used in clinical practice. This method involves dividing the lung horizontally at the lower boundaries of the second and fourth ribs, resulting in 3 distinct fields: upper, middle, and lower. Additionally, the lung is vertically partitioned into inner, middle, and outer bands, generating a total of 18 partitions across both left and right lungs. Two additional partitions representing the mediastinum and lateral images are included, summing up to 20 partitions in total, aligning with established clinical standards [63]. In our study, we employed the 20-partition method to interpret the model results. Model performance was assessed using metrics such as the AUC, accuracy, and recall. AUC values were computed for each of the 20 partitions across 26 diagnoses to elucidate key diagnostic trends (refer to Figs. S1 to S7 for the partitioning method). To enhance interpretability, Grad-CAM was employed to generate post hoc explanations, specifically coarse localization heatmaps highlighting pivotal regions in chest radiographs for diagnosis prediction [31]. Grad-CAM has been established as a robust method for elucidating deep learning models, contributing to the explainability of our CNN model [64,65]. Representative images were carefully selected and presented in the Results section to provide visual context and further illustrate our findings.

### Accuracy comparison between the CNN model and physicians

To assess the diagnostic accuracy of both physicians and the CNN model in interpreting chest radiographs, we enlisted the participation of 16 registered physicians from 7 hospitals. This cohort included specialists in cardiology, respiratory medicine, and thoracic surgery, each with a minimum of 3 years of experience in their respective fields. Additional details regarding the physicians’ backgrounds are available in the Supplementary Materials for reference.

### Generalizability evaluation and cognitive ability of the CNN model

To evaluate the reproducibility and generalizability of our CNN algorithm across datasets, a total of 313 lung opacity chest radiographs were obtained between 2023 January 6 and 2023 March 30 from Chinese PLA General Hospital. Lung opacity was chosen as the focus due to its prevalence among chest radiographs of respiratory inpatients during the study timeframe. Three board-certified thoracic radiologists reviewed all chest radiographs, independently documenting their radiographic findings, with consensus diagnoses serving as ground truths. The model’s performance on the 2 local external datasets were evaluated based on AUC values.

To test the model’s learning capabilities, we used a local dataset comprising 20 post-TAVI chest radiographs and 5 normal chest radiographs. Given the absence of TAVI chest radiographs in the training and testing datasets, this step aimed to test the large AI’s capacity for self-learning. The dataset was sourced from Fuwai Yunnan Cardiovascular Hospital.

### Trans-device image recognition

The CNN model’s diagnostic proficiency on images from different devices was tested using Photo-DEV dataset, encompassing 202 photographs corresponding to the testing dataset provided by CheXpert. These photographs were captured 10 times by a single physician. During the initial 9 captures, varying device settings were employed under identical lighting conditions and spatial settings. Three different smartphones (Apple iPhone 6s, Acer Z330, and Asus Zenfone 5z) and 3 different computer monitors (MSI GE 40, Dell SE2417HGX, and Asus VE278) were utilized to manipulate device settings. An additional set of 202 photographs was collected under brighter lighting conditions, culminating in a total of 2020 photographs for the Photo-DEV dataset [46,47]. This dataset was meticulously chosen to simulate diverse methodologies for acquiring chest radiographs, thereby evaluating our model’s performance in complex environments. We intersected our dataset with these data and included 8 diagnoses that were shared between the 2 datasets for testing, including cardiomegaly, oedema, lung lesion, lung opacity, no finding or normal, pneumonia, pneumothorax, and support devices.

### Application of the CNN-based model for untrained diagnoses and its optimization of the medical process

Although we believe that the 26 diagnoses included in the training model already cover most of the common clinical diseases and far exceed those included in similar studies [23,66], it is still impossible to include all types of diseases. Therefore, clinical decisions from human experts are still necessary. However, our model can provide basic descriptions of diseases such as “Effusion OR Pleural Effusion,” “Enlarged Cardiomediastinum,” and “Lung Opacity,” aiding clinicians in promptly and accurately identifying patients with abnormal chest radiographs. This proves especially crucial for individuals with urgent conditions, where overlooking a diagnosis could lead to serious consequences. For instance, in cases of aortic dissection, many primary medical institutions lack the capability to perform enhanced computed tomography (CT) scans for diagnosis. Thus, the ability of chest radiographs to aid in identifying such patients holds significant clinical value. To validate this assertion, we included 70 consecutive patients (aged >19 years) clinically diagnosed with aortic dissection by physicians at PLA General Hospital from January to March 2022. Please refer to Table S1 for a description of patient baseline data. Chest radiographs were obtained upon admission. We engaged 8 experts, including senior and junior experienced radiologists as well as nonradiological physicians, with 2 experts allocated to each of the 4 groups. In accordance with the established models, the physicians were obligated to choose from the 26 labels. Subsequently, after an initial independent interpretation, the 8 experts reread the chest radiographs 2 months later with AI assistance. The accuracy of both interpretations was compared and analyzed, employing the paired McNemar test to assess consistency across different groups. As the diagnosis of aortic dissection is not one of the 26 label categories, expert judgement was required solely to determine whether this chest radiograph was abnormal and required further investigation.

### Fairness analysis of the CNN model

There have been documented concerns in the literature regarding potential biases and diminished performance quality of AI models in populations with limited medical resources, particularly among female patients [67]. Hence, ensuring the fairness of AI models across diverse populations, especially between males and females, is paramount. To verify whether discrepancies exist in our model’s performance between male and female populations, we conducted AI fairness validation on chest radiographs diagnosed with “Effusion OR Pleural Effusion,” “Pneumothorax,” and “Lung Opacity.” We specifically chose these 3 labels due to their representation of categories demonstrating the poorest, second poorest, and median AUC performance of the model on the validation set, respectively. For “Effusion OR Pleural Effusion” and “Pneumothorax,” we included chest radiographs of respiratory department inpatients from the PLA General Hospital, including 165 and 118 cases, respectively, documented from January to March 2022. “Lung Opacity” used the previously collected dataset, which included 313 patients. Please refer to Table S1 for a description of patient baseline data.

### Development of web-based software application

In order to disseminate our model to the intended users, the proposed model was successfully integrated into a robust real-world system. Additionally, a user-friendly application was developed to facilitate the utilization of the model, which can be accessed at the following URL: https://tfpic.benefm.com/ (please refer to the Supplementary Materials for additional details).

### Statistical analysis

The development of the CNN PanCV model was performed using Python 3.9, a widely used programming language for scientific computing and data analysis [68]. To visualize the results of the PanCV model, we used the Matplotlib package, which is a powerful and flexible data visualization library in Python [69]. All statistical analyses were performed using R statistical software (version 3.5.1) [70]. Receiver operating characteristic (ROC) analyses and jackknife alternative free-response ROC (JAFROC) analyses were conducted to assess the performances of image classification and lesion localization, respectively. All results with 2-sided *P* values less than 0.05 were considered statistically significance, and the Holm–Bonferroni method was applied to correct for multiple comparisons.

## Data Availability

The study utilized a total of 13 datasets, among which 11 are openly available and can be accessed as depicted in Table 1. The datasets from the Fuwai Yunnan Cardiovascular Hospital and the PLA General Hospital can be obtained by contacting the corresponding author (panxiangbin@fuwaihospital.org) and confirming that they will only be used for academic research purposes. All software tools and related codes are open source and can be found at: https://tfpic.benefm.com/.

## Supplementary Materials

Supplementary 1 Supplementary Methods Figs. S1 to S16 Tables S1 to S7 References
