# Supplementary material for: Development and External Validation of an Artificial Intelligence-Based Method for Scalable Chest Radiograph Diagnosis: A Multi-Country Cross-Sectional Study
Source: Research (Wash D C). 2024 Aug 6;7:0426. doi: 10.34133/research.0426 (PMC11301699; doi:10.34133/research.0426)
Supplement: Supplementary 1 — Supplementary Methods Figs. S1 to S16 Tables S1 to S7 References [file research.0426.f1.pdf]

Supplementary material

Development and external validation of artificial intelligence-based method for scalable chest radiograph diagnosis for surgery: a multi-country cross-sectional study

This PDF file includes:

Supplementary methods

Supplementary Figures S1 to S16

Figure S1. Results of the upper, middle, and lower fields in the outer band of the right lung

Figure S2. Results of the upper, middle, and lower fields in the middle band of the right lung

Figure S3. Results of the upper, middle, and lower fields in the inner band of the right lung

Figure S4. Results of the upper, middle, and lower fields in the inner band of the left lung

Figure S5. Results of the upper, middle, and lower fields in the middle band of the left lung

Figure S6. Results of the upper, middle, and lower fields in the outer band of the left lung

Figure S7. Results of cardiac region and the lateral image

Figure S8. Software-generated heatmap visualization results (AorticEnlargement, Atelectasis, Calcification)

Figure S9. Software-generated heatmap visualization results (Cardiomegaly, Consolidation, COVID- 19)

Figure S10. Software-generated heatmap visualization results (Edema, EffusionORPleuralEffusion, Emphysema)

Figure S11. Software-generated heatmap visualization results (EnlargedCardiomediastinum, EnlargedPA, Fibrosis)

Figure S12. Software-generated heatmap visualization results (Fracture, Hernia, Infiltration)

Figure S13. Software-generated heatmap visualization results (LungLesion, LungOpacity, Mass)

Figure S14. Software-generated heatmap visualization results (MediastinalShift, Nodule, NoFindingorNormal)

Figure S15. Software-generated heatmap visualization results(PleuralOther, Pneumonia, Pneumothorax)

Figure S16. Software-generated heatmap visualization results (SupportDevices, tuberculosis) and the software interface (C).

Supplementary Table S1 to S2

Table S1. Local data information table (this table contains all patient information from PLA General Hospital and Fuwai hospital)

Table S2. The number of chest radiographs included in each label after reorganization.

Table S3. Model hyper-parameter details.

Table S4. Model training details.

Table S5. Summary of additional model evaluation metrics.

Table S6: Innovations and improvements of the model.

Table S7. Summary of other related similar studies.

Supplementary methods

Development of the CNN diagnostic algorithm

The CNN models can adeptly identify low-, medium-, and high-level features from images, and stacking more layers usually increases the accuracy of the model. The emergence of ResNet has solved the problem of gradient explosion when the network becomes more complex and has substantial advantages in processing large-scale datasets. Therefore, we developed our model on the ResNet50 architecture, replacing the last layer of the model by changing the activation function of the dense fully connected layer to sigmoid. In the multinational multicentre datasets used here, there were differences in the chest radiograph standards and the distribution of labels. To enable the model to learn the features of different datasets stably and efficiently, we employed several methods for input standardization data augmentation, including image flipping and colour space transformation. Finally, we scaled all images to a 512 x 512 pixel size. To better capture the features of different labels, we modelled each output as an independent binomial distribution.

Accuracy comparison between the CNN model andphysicians

Specifically, the participants were from the following hospitals: the Chinese Academy of Medical Sciences, Fuwai Hospital (1 cardiologist); the People's Hospital of Tibet Autonomous Region (1 cardiologist); Gansu Provincial Hospital (1 cardiologist); Fuwai Yunnan Cardiovascular Hospital (8 radiologists); Peking Union Medical College Hospital (1 pulmonologist); Peking University Third Hospital (1 pulmonologist); Central China Fuwai Hospital (1 cardiologist); and the Chinese Academy of Medical Sciences, Cancer Hospital (2 thoracic surgeons). The diagnoses with the best, worst, and median AUC values in the testing set, COVID-19, pneumothorax, and effusion or pleural effusion, respectively, were selected for this comparison. Fifty images corresponding to each of these three diagnoses were randomly selected from the testing set and given to human experts for evaluation. The accuracy of the human expert's evaluation was then compared to that of the machine's evaluation. To ensure fairness in the comparison, the human experts were provided with training data in advance.

Development of web-based software application

The model server module, powered by tf-serving\footnote{https://github.com/TensorFlow/serving}, plays a critical role in predicting the probability of each disease. Leveraging state-of-the-art algorithms and techniques, it precisely analyses the input data, enabling the generation of accurate disease predictions. Conversely, the logic module, built on Flask\footnote{https://flask.palletsprojects.com/}, acts as an intermediary between the front-end page and the model server module. Whenever a user inputs a chest radiograph, the logic module processes the image, transforming it into a matrix, which is then seamlessly fed into the model server module for prediction. Following the retrieval of the output probability, the logic module effectively identifies and returns the top-K (e.g., 5) diseases with the highest probabilities.

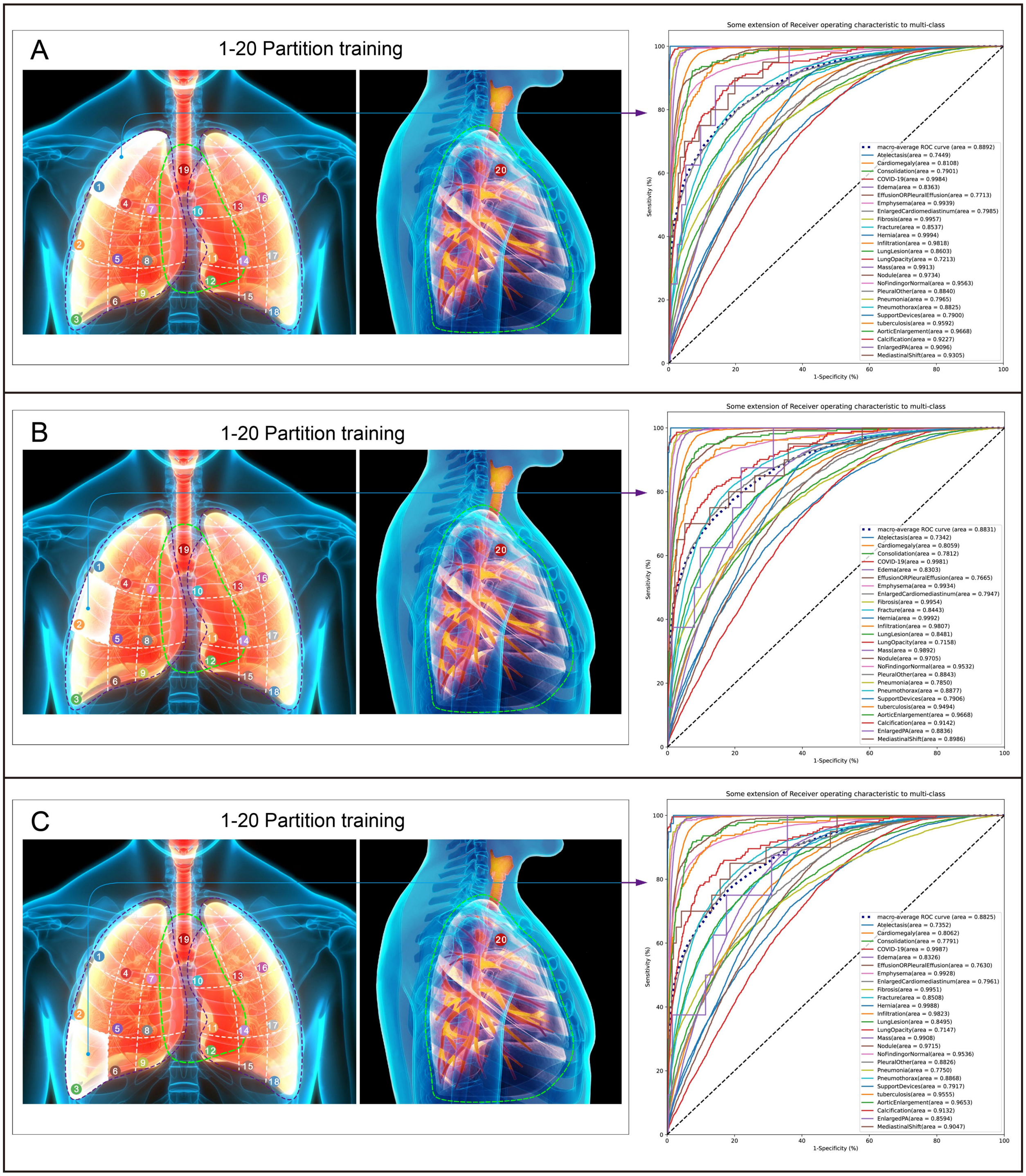

Figure S1. Results of the upper (A), middle (B), and lower (C) fields in the outer band of the right lung

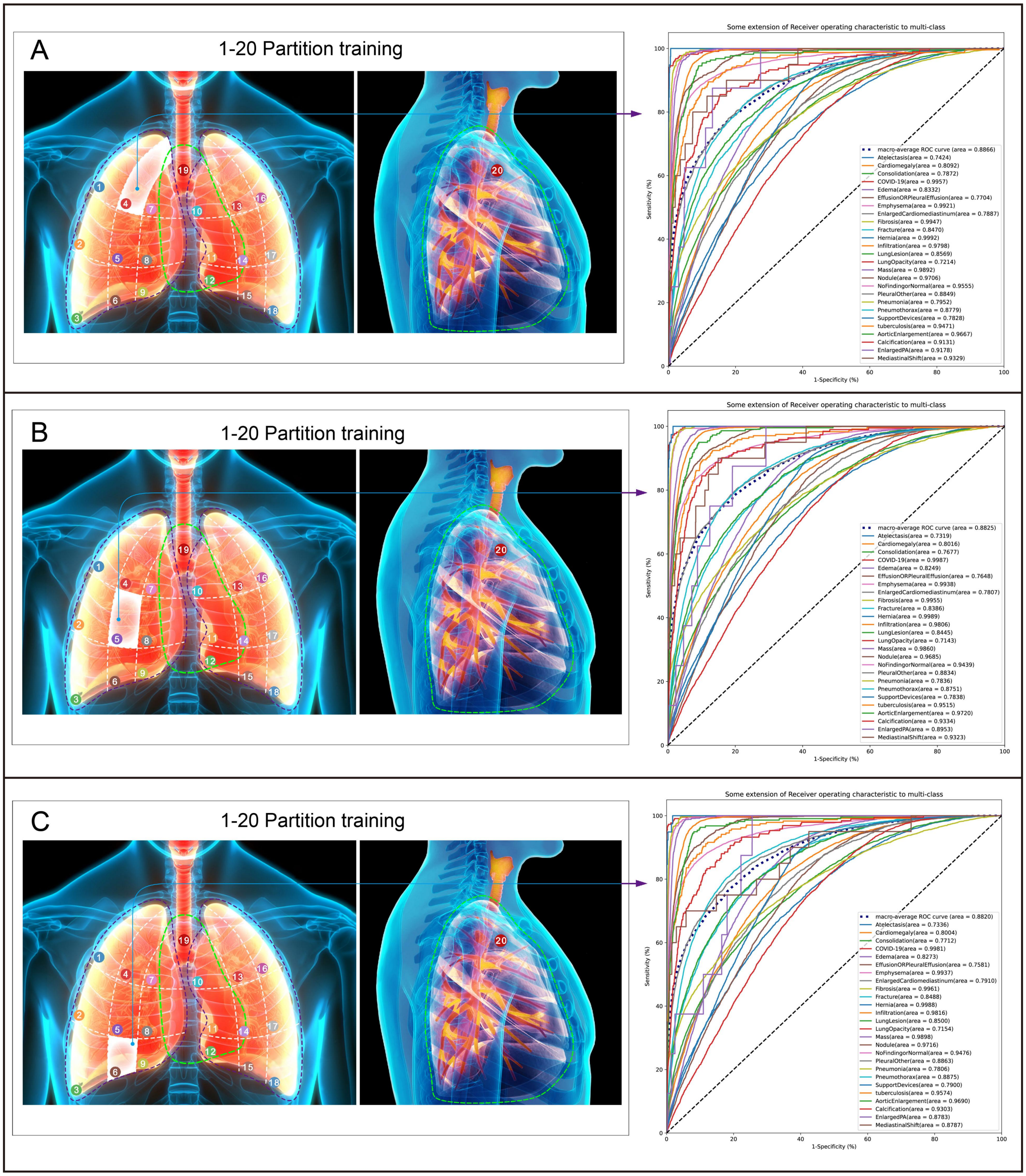

Figure S2. Results of the upper (A), middle (B), and lower (C) fields in the middle band of the right lung

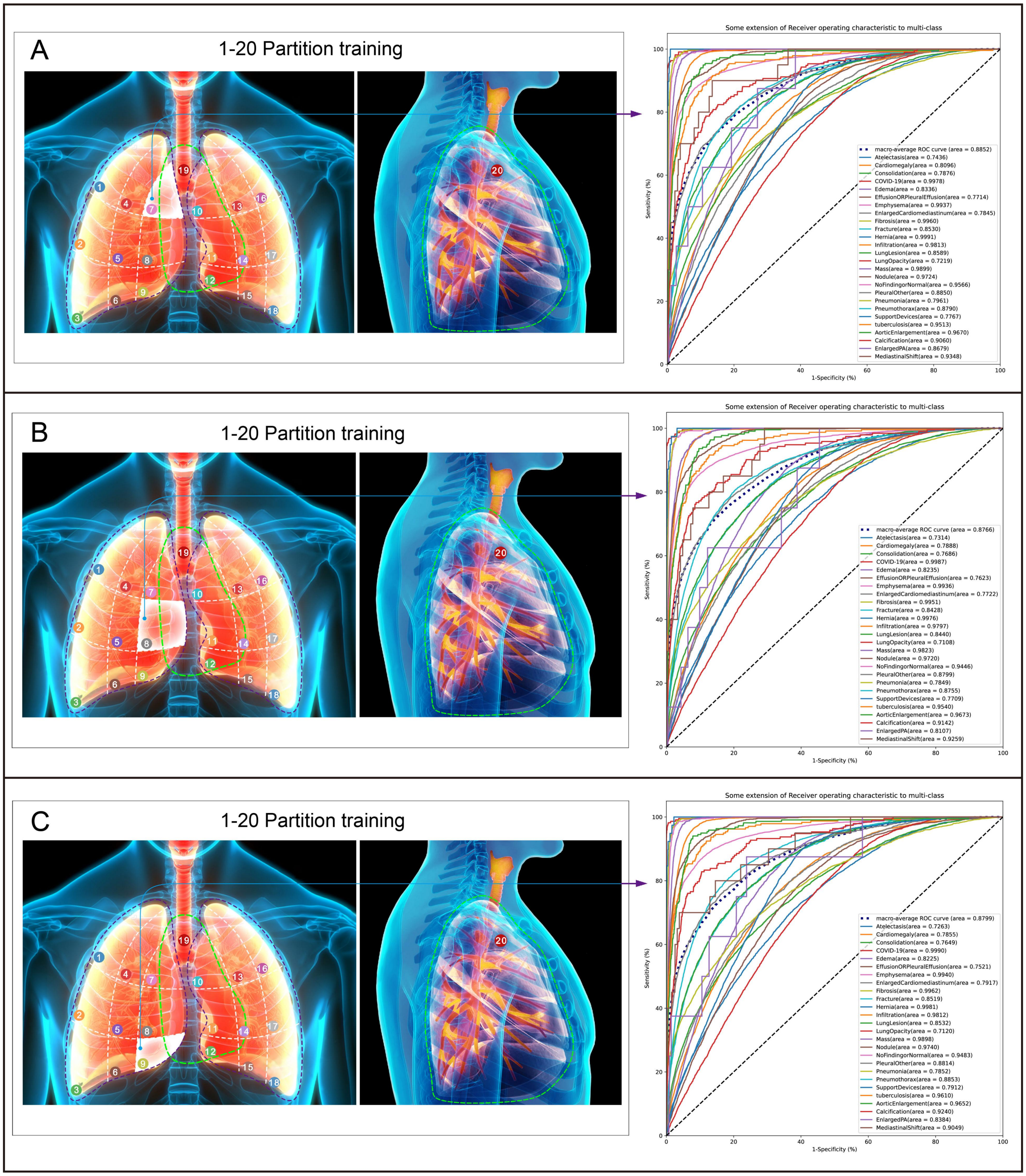

Figure S3. Results of the upper (A), middle (B), and lower (C) fields in the inner band of the right lung

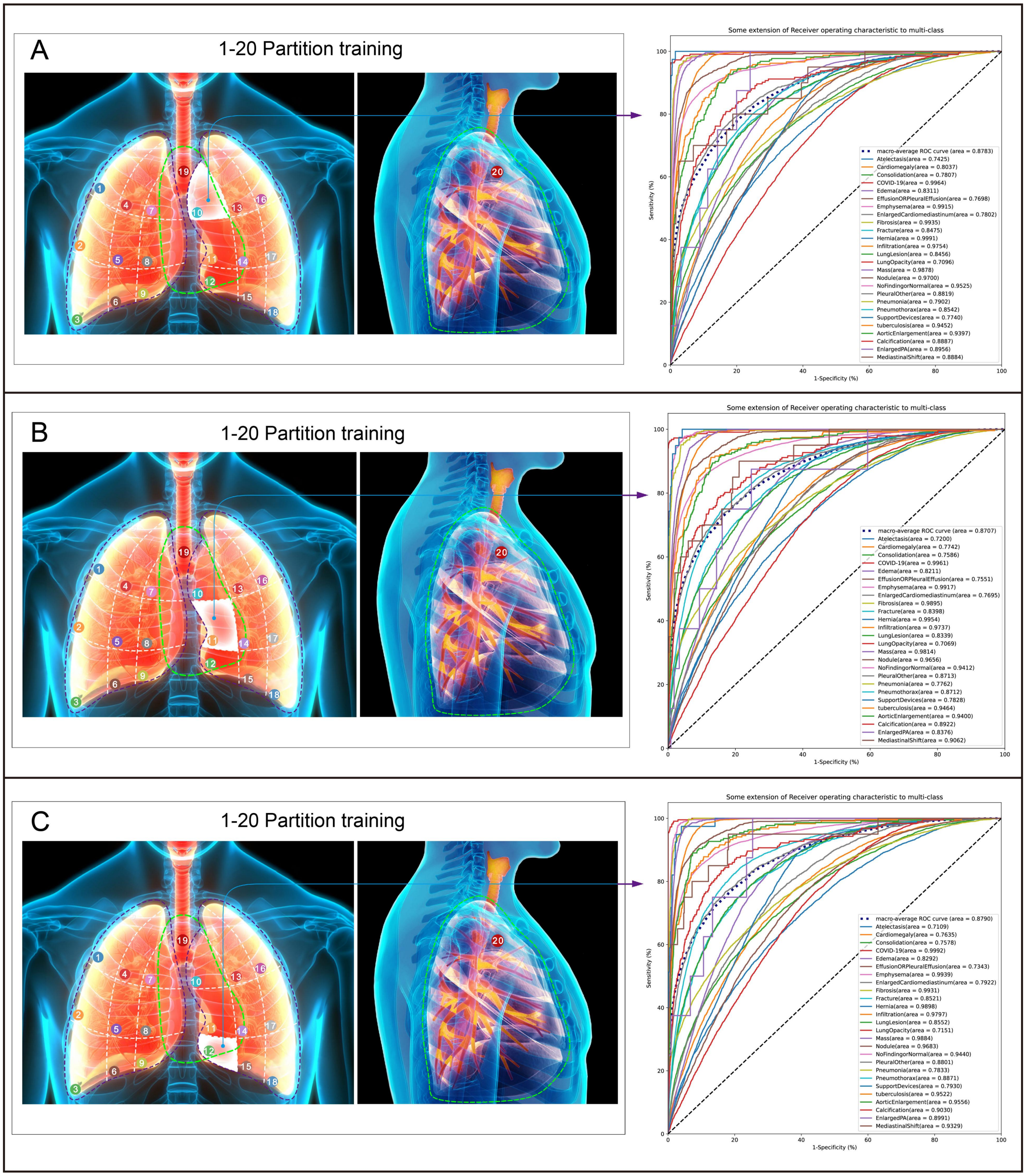

Figure S4. Results of the upper (A), middle (B), and lower (C) fields in the inner band of the left lung

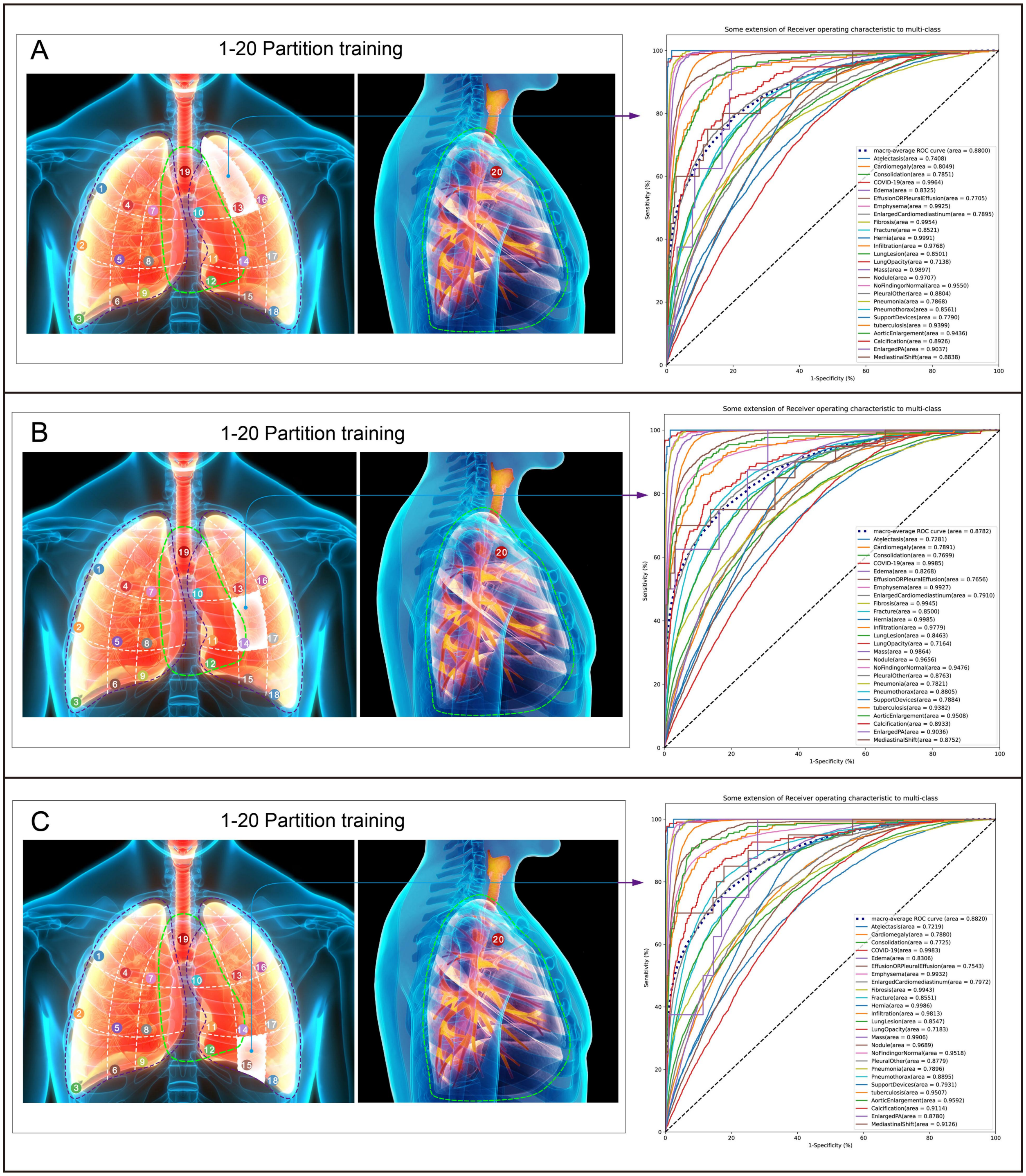

Figure S5. Results of the upper (A), middle (B), and lower (C) fields in the middle band of the left lung

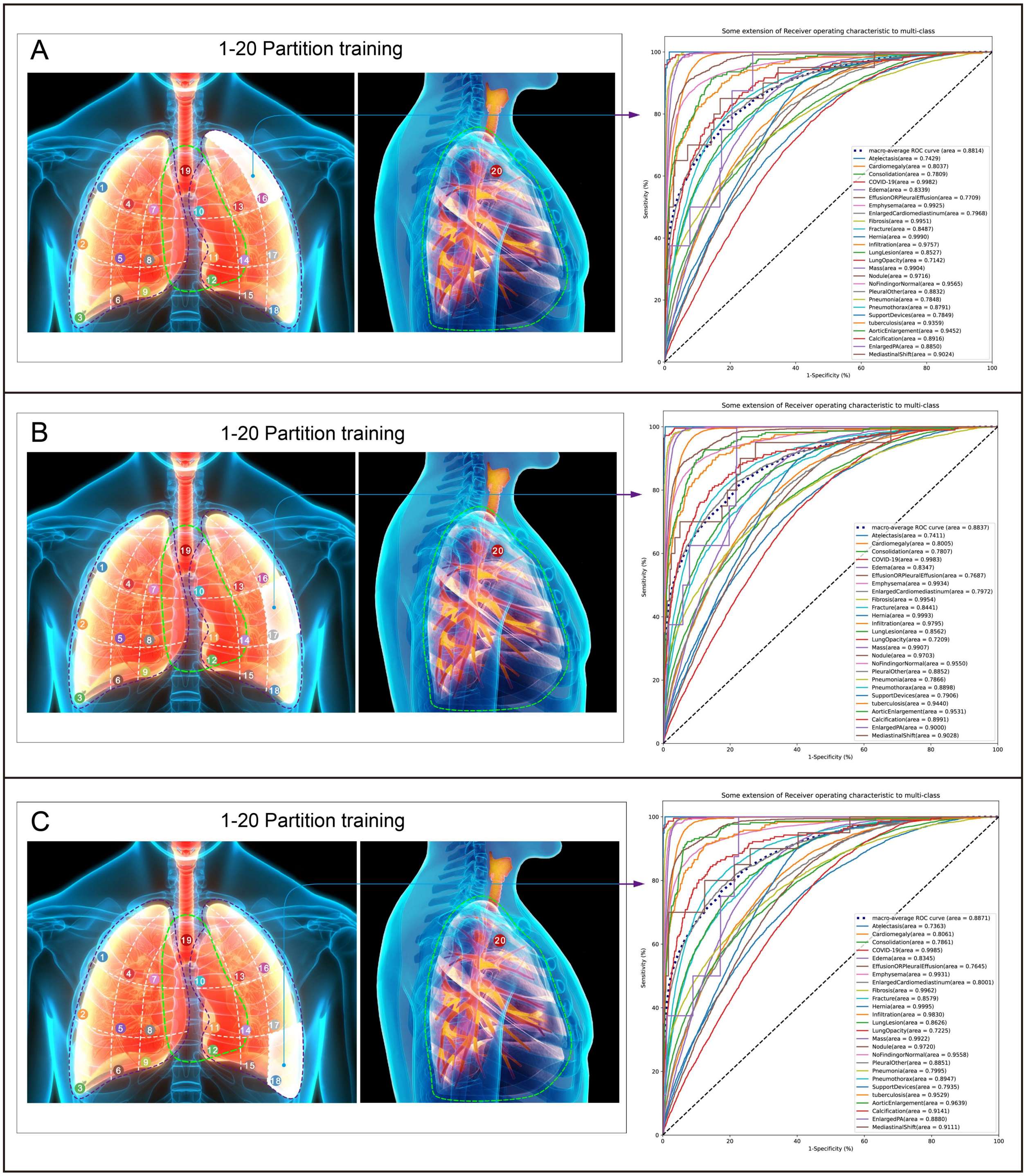

Figure S6. Results of the upper (A), middle (B), and lower (C) fields in the outer band of the left lung

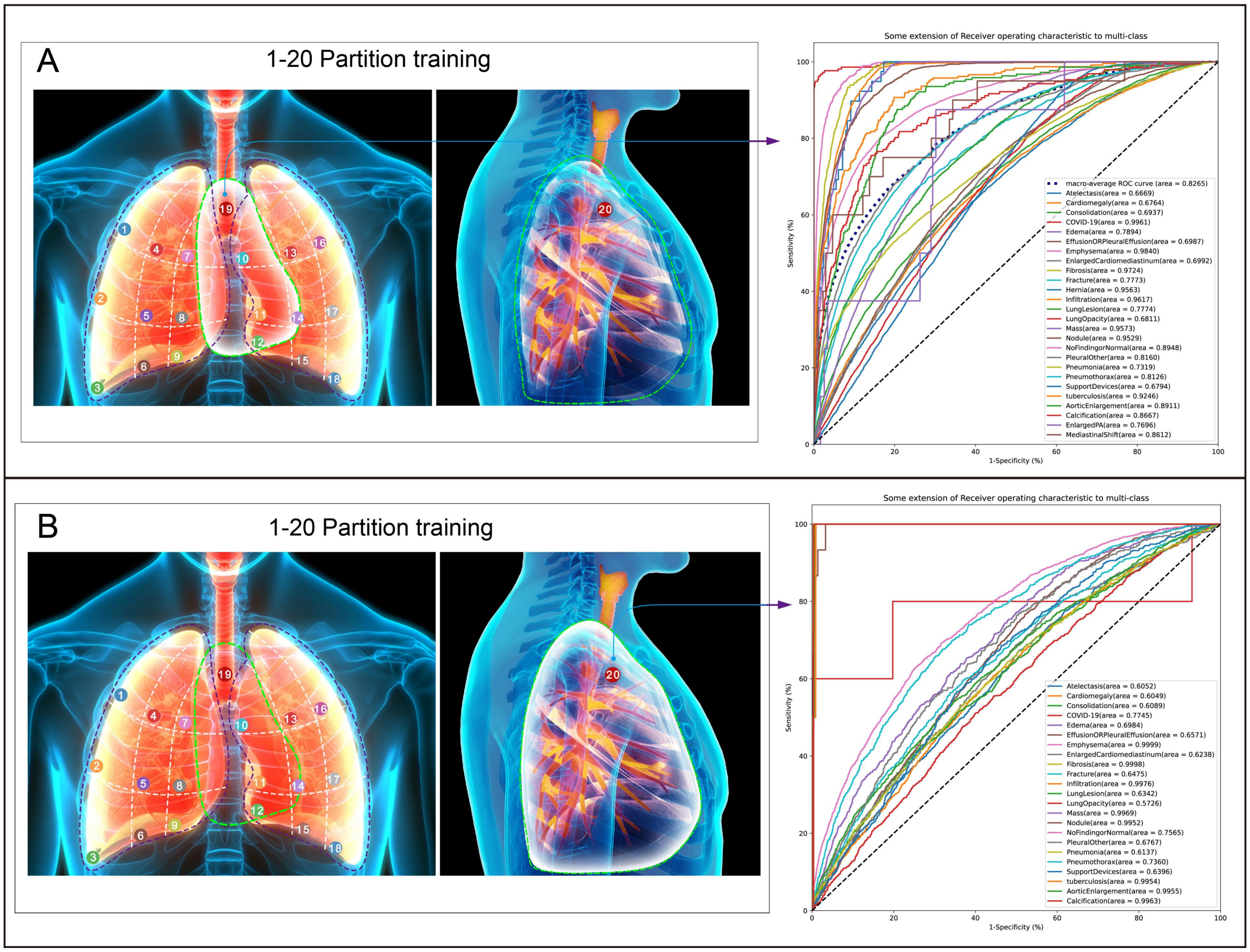

Figure S7. Results of cardiac region (A) and the lateral image (B)

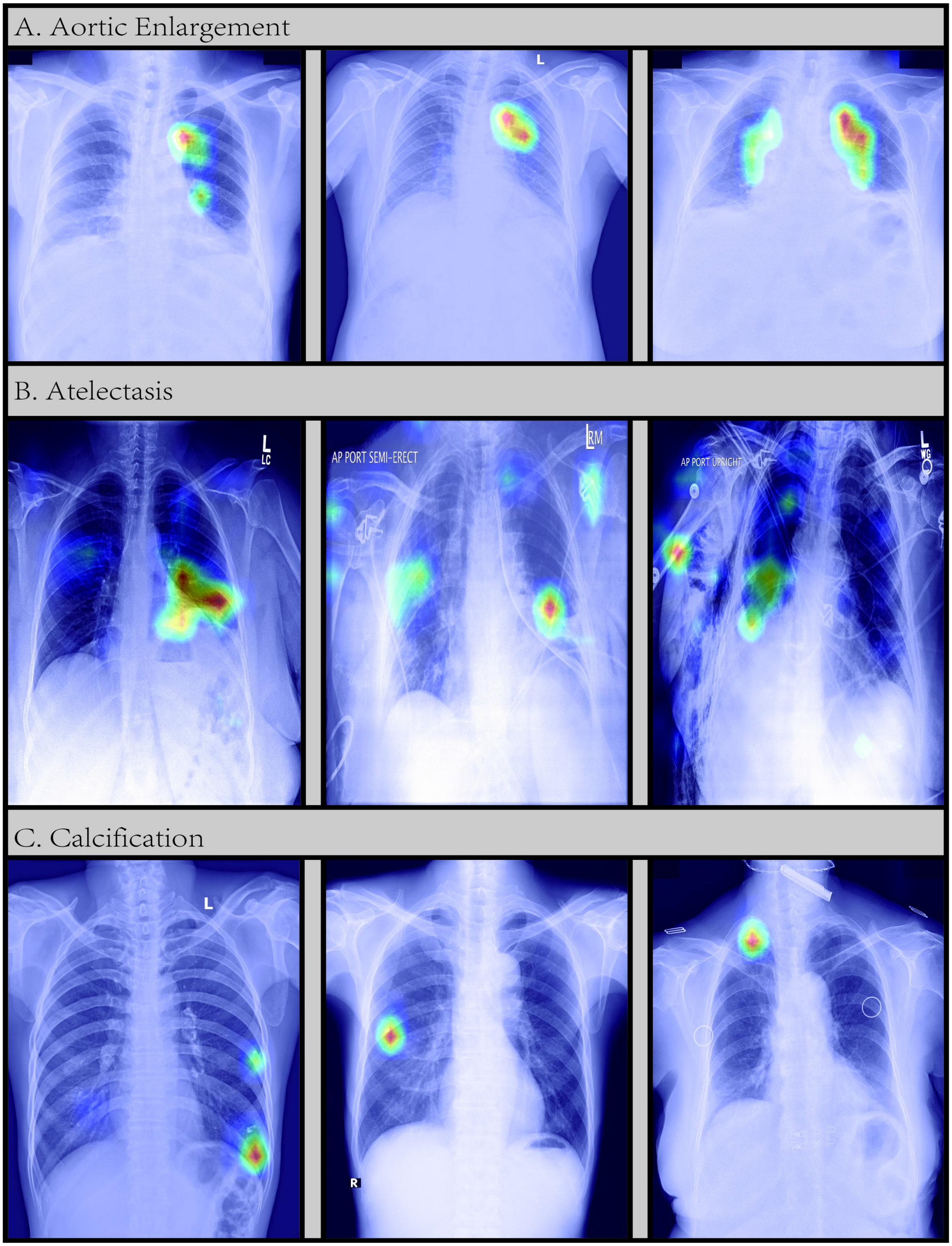

Figure S8. Software-generated heatmap visualization results (AorticEnlargement, Atelectasis, Calcification)

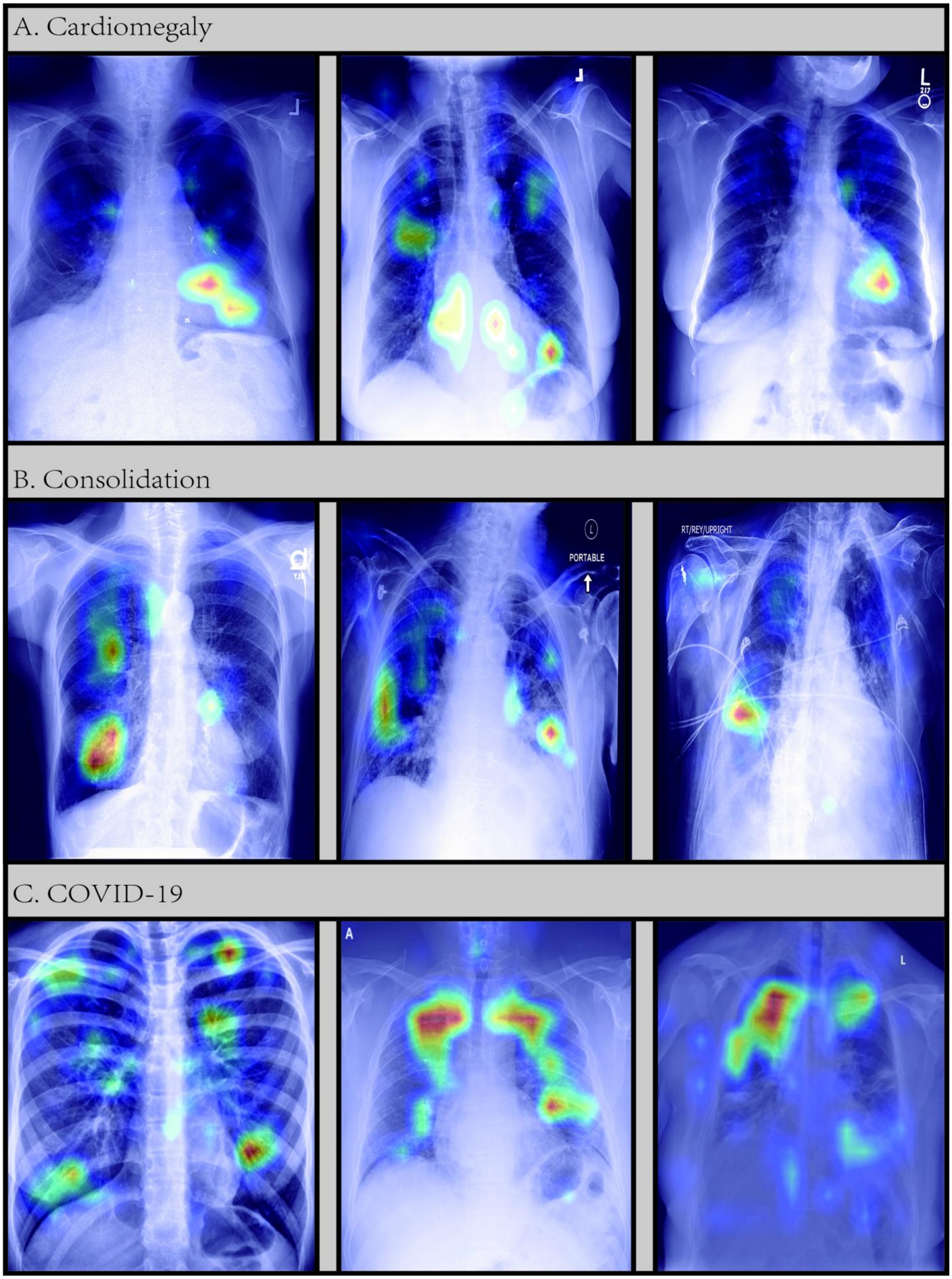

Figure S9. Software-generated heatmap visualization results (Cardiomegaly, Consolidation, COVID-19)

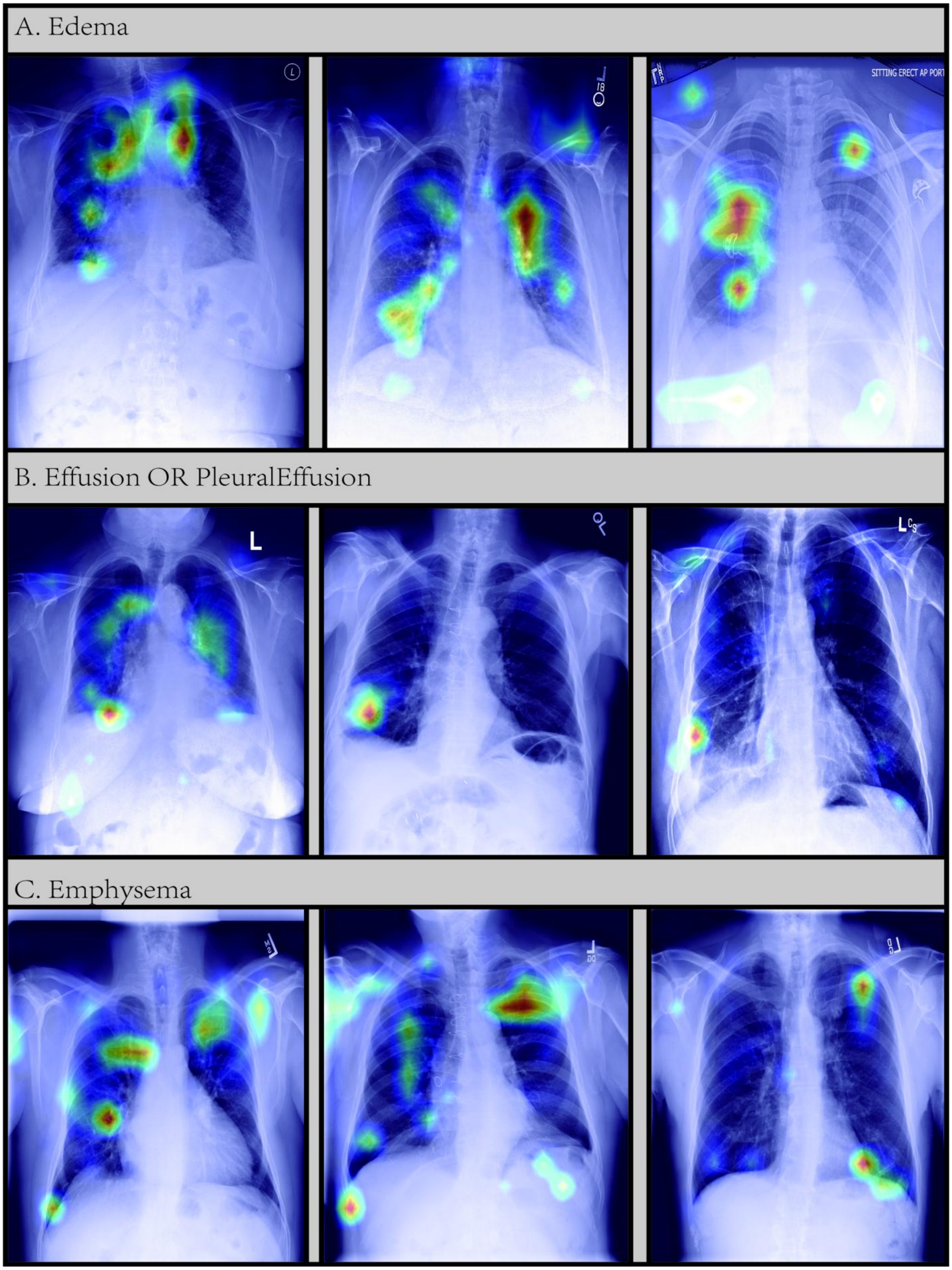

Figure S10. Software-generated heatmap visualization results (Edema, EffusionORPleuralEffusion, Emphysema)

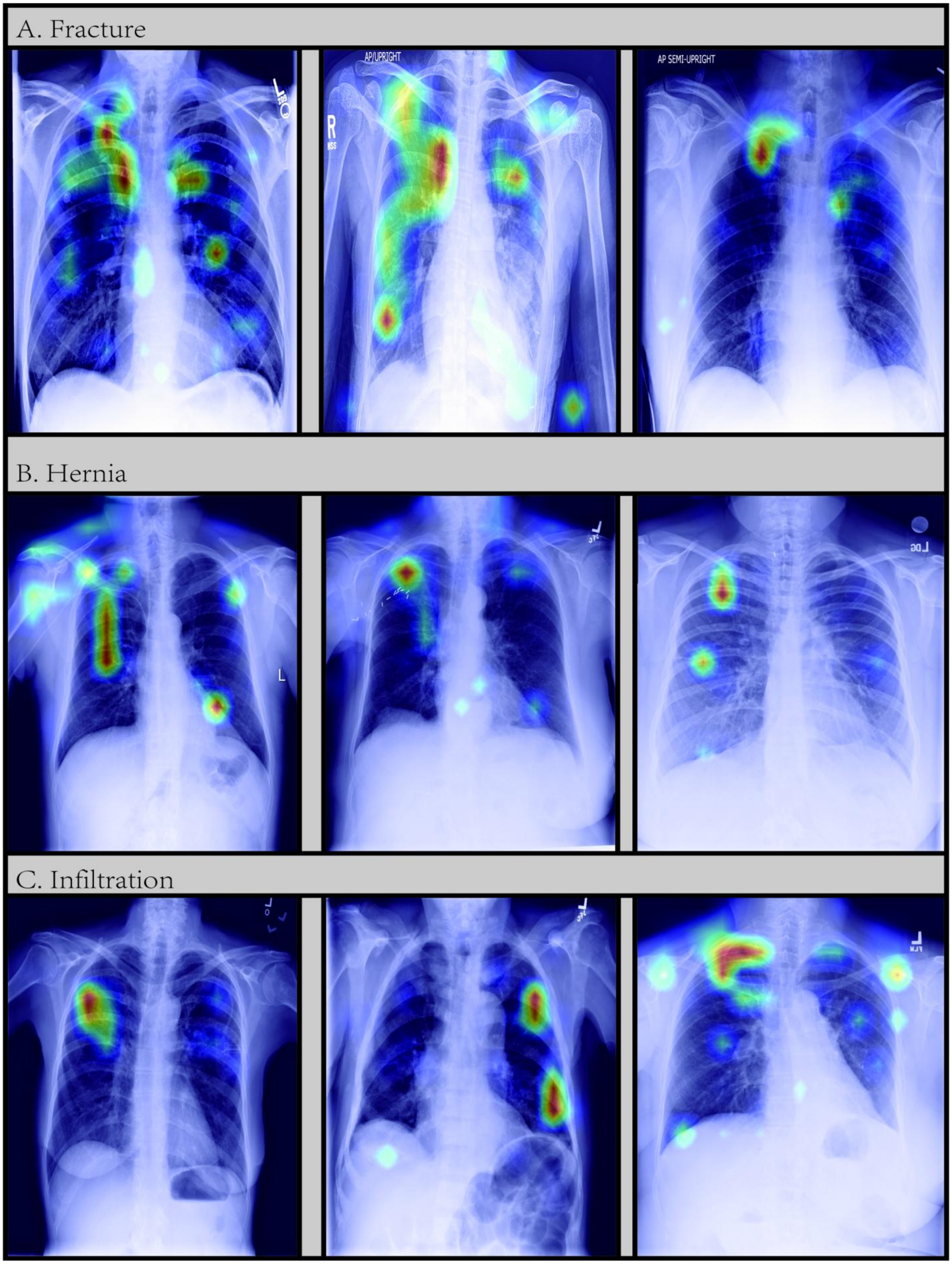

Figure S12. Software-generated heatmap visualization results (Fracture, Hernia, Infiltration)

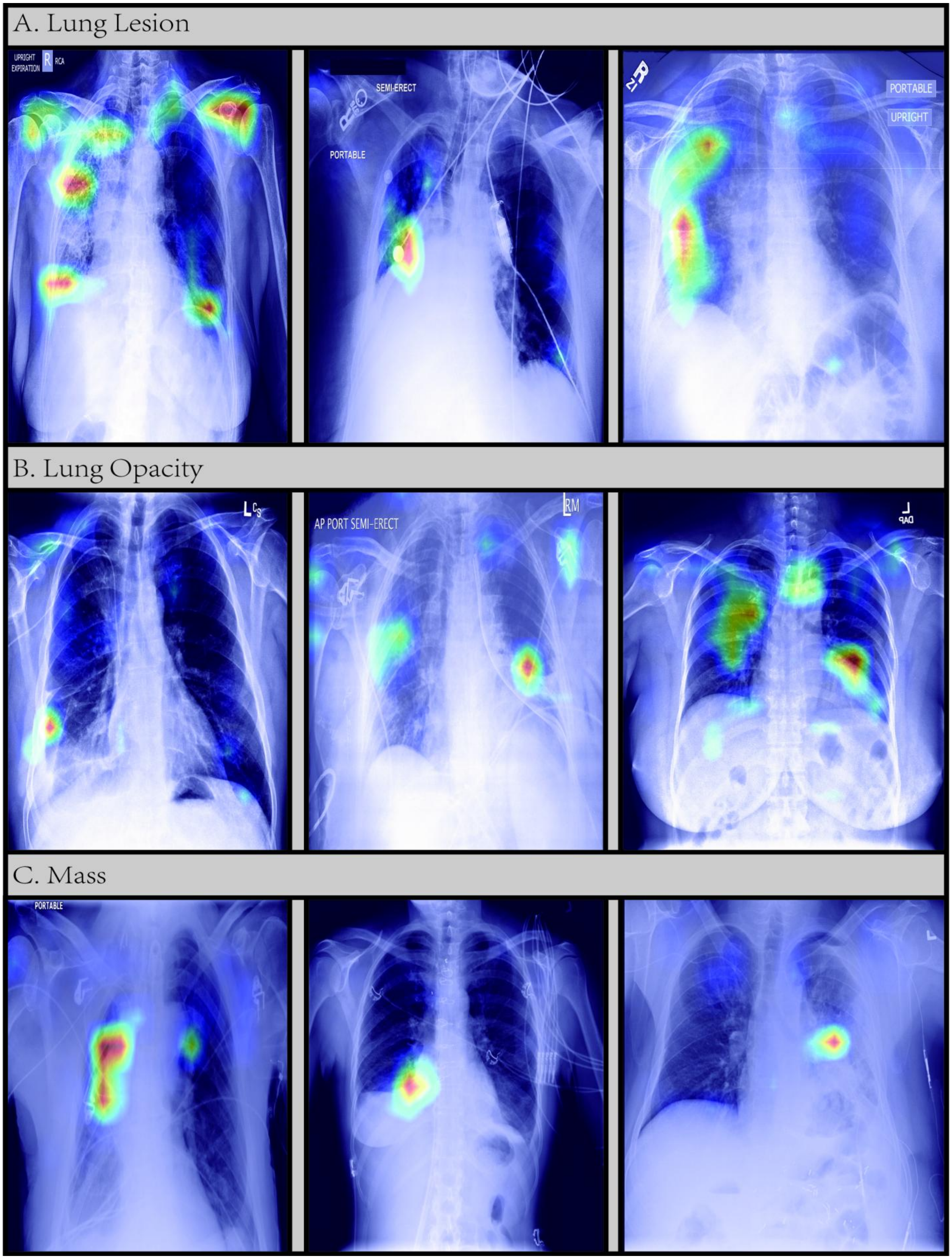

Figure S13. Software-generated heatmap visualization results (LungLesion, LungOpacity, Mass)

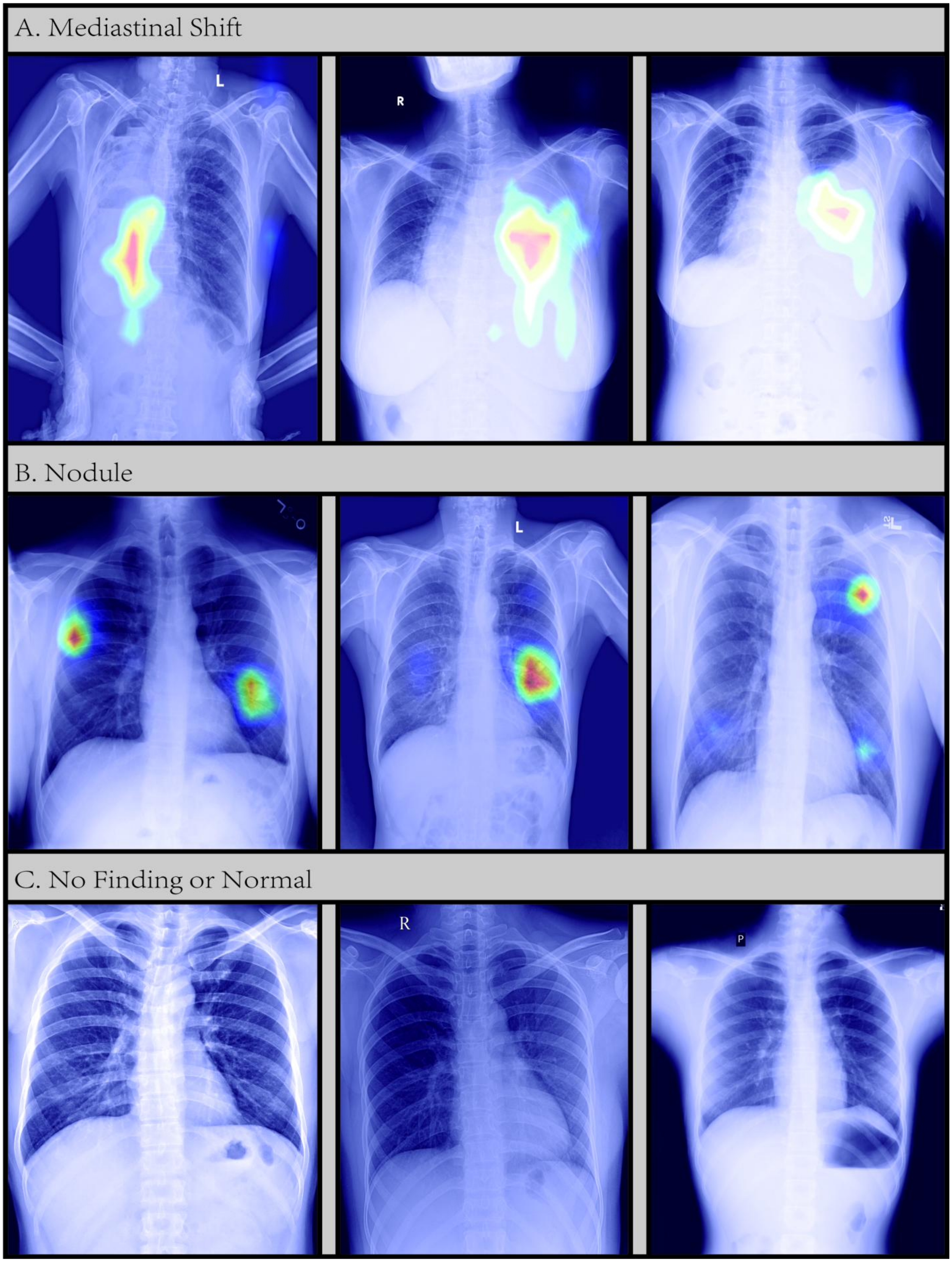

Figure S14. Software-generated heatmap visualization results (MediastinalShift, Nodule, NoFindingorNormal)

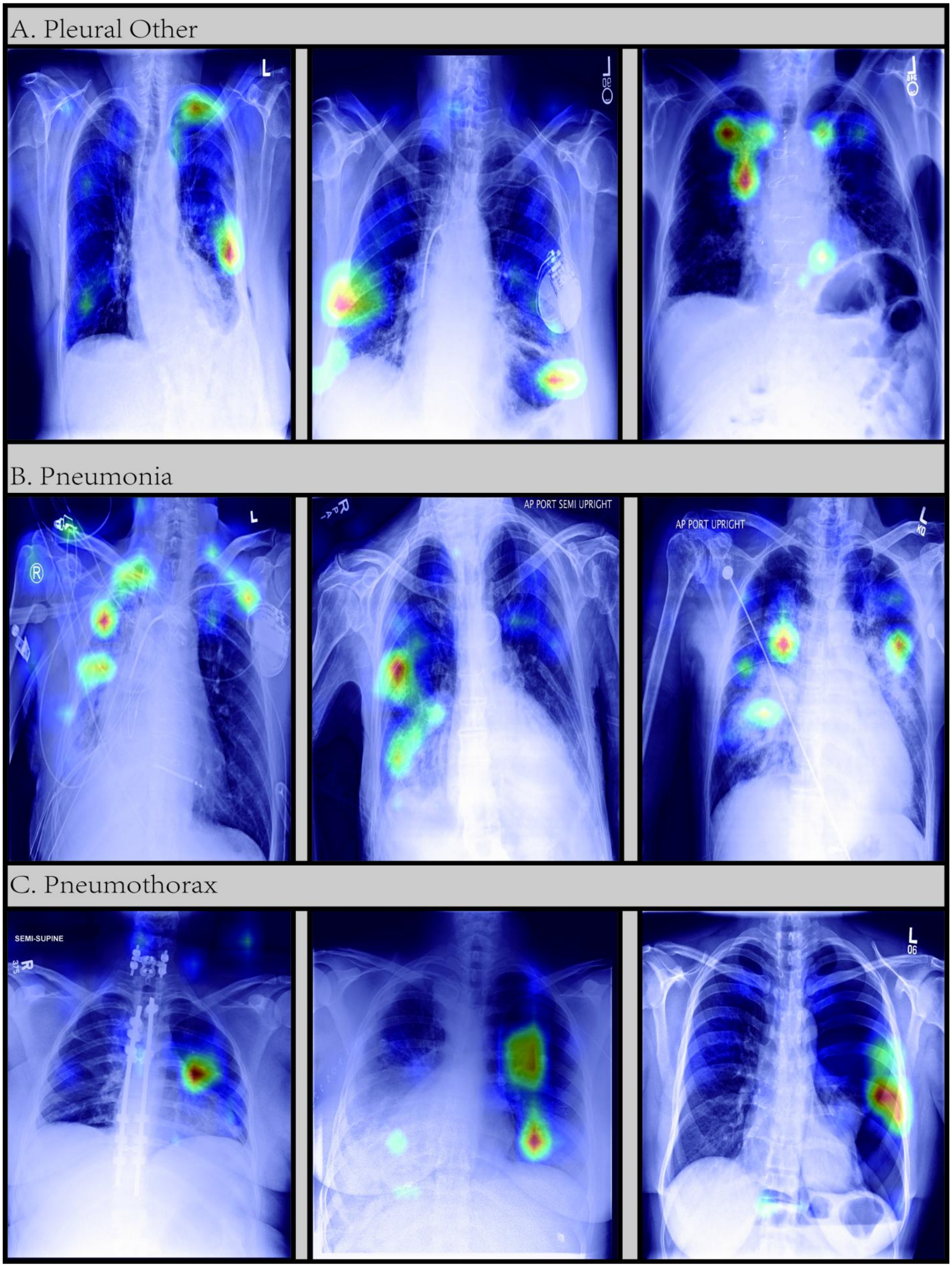

Figure S15. Software-generated heatmap visualization results(PleuralOther, Pneumonia, Pneumothorax)

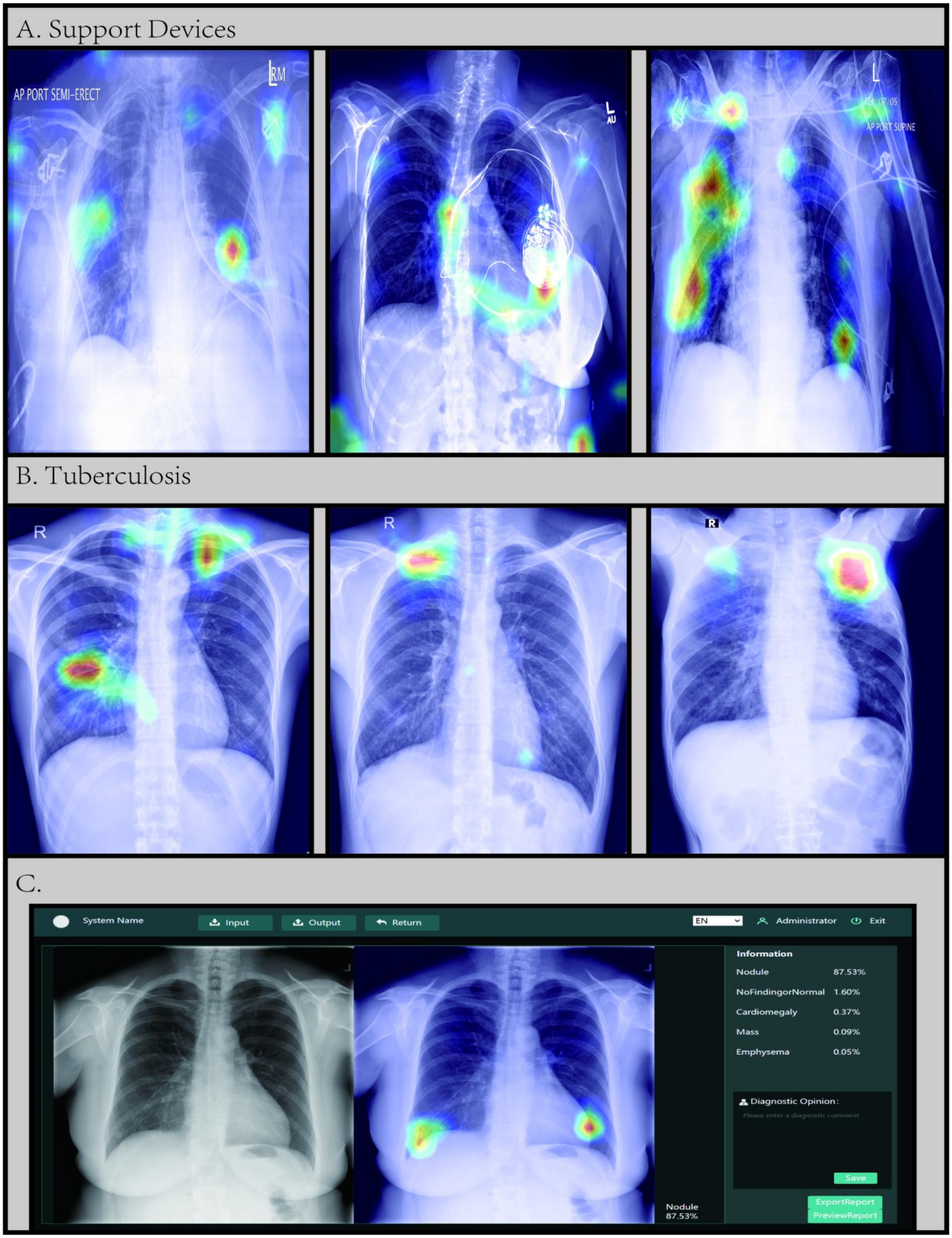

Figure S16. Software-generated heatmap visualization results (SupportDevices, tuberculosis) and the software interface (C).

Table S1. Local data information table (this table contains all patient information from PLA General Hospital and Fuwai hospital)

Research Population	Characteristic	
**Lung Opacity (n=313)-PLA**		**Mean+SD**
	**Age, y**	85.4 ± 7.9
	**Height, cm**	159.5 ± 7.6
	**Weight, kg**	60.0 ± 11.1
		N (%)
	**Sex**	
	female	241 (77.0%)
	male	72 (23.0%)
	**Corory heart disease**	
	without this disease	156 (49.8%)
	with this disease	157 (50.2%)
	**Hypertension**	
	without this disease	122 (39.0%)
	with this disease	191 (61.0%)
	**Cerebral infarction or cerebral hemorrhage**	
	without this disease	252 (80.5%)
	sickness	61 (19.5%)
	**Diabetes**	
	without this disease	240 (76.7%)
	with this disease	73 (23.3%)
**Effusion OR Pleural Effusion (n=165)-PLA**		
		Mean+SD
	**Age, y**	74.5 ± 17.4
	**Height, cm**	166.1 ± 8.1
	**Weight, kg**	63.9 ± 12.7
		N (%)
	**Sex**	
	female	64 (38.8%)
	male	101 (61.2%)
	**Corory heart disease**	
	without this disease	130 (78.8%)
	with this disease	35 (21.2%)
	**Hypertension**	

	without this disease	110 (66.7%)
	with this disease	55 (33.3%)
	**Cerebral infarction or cerebral hemorrhage**	
	without this disease	151 (91.5%)
	with this disease	14 (8.5%)
	**Diabetes**	
	without this disease	128 (77.6%)
	with this disease	37 (22.4%)
**Pneumothorax (n=118)-PLA**		
		Mean+SD
	**Age, y**	57.2 ± 15.9
	**Height, cm**	164.9 ± 12.8
	**Weight, kg**	62.0 ± 12.1
		N (%)
	**Sex**	
	female	61 (51.7%)
	male	57 (48.3%)
	**Corory heart disease**	
	without this disease	105 (89.0%)
	with this disease	13 (11.0%)
	**Hypertension**	
	without this disease	96 (81.4%)
	with this disease	22 (18.6%)
	**Cerebral infarction or cerebral hemorrhage**	
	without this disease	113 (95.8%)
	with this disease	5 (4.2%)
	**Diabetes**	
	without this disease	108 (91.5%)
	with this disease	10 (8.5%)
**Aortic Dissection (n=70)-PLA**		
		Mean+SD
	**Age, y**	50.6 ± 13.3
	**Height, cm**	171.9 ± 7.0
	**Weight, kg**	78.3 ± 13.6
		N (%)
	**Sex**	
	female	41 (58.6%)
	male	29 (41.4%)
	**Corory heart disease**	

	without this disease	64 (91.4%)
	with this disease	6 (8.6%)
	**Hypertension**	
	without this disease	27 (38.6%)
	with this disease	43 (61.4%)
	**Cerebral infarction or cerebral hemorrhage**	0.0 ± 0.0
	**Diabetes**	0.0 ± 0.0
**Normal (n=165)-PLA**		
		Mean+SD
	**Age, y**	47.1 ± 16.1
	**Height, cm**	165.6 ± 11.5
	**Weight, kg**	67.9 ± 17.5
		N (%)
	**Sex**	
	female	80 (48.5%)
	male	85 (51.5%)
	**Corory heart disease**	
	without this disease	158 (95.8%)
	with this disease	7 (4.2%)
	**Hypertension**	
	without this disease	142 (86.1%)
	with this disease	23 (13.9%)
	**Diabetes**	
	without this disease	159 (96.4%)
	with this disease	6 (3.6%)
	**Cerebral infarction or cerebral hemorrhage**	0.0 ± 0.0
**TAVI (n=18)-Fuwai**		
		Mean+SD
	**Age, y**	71.4 ± 9.3
	**Height, cm**	161.4 ± 9.9
	**Weight, kg**	58.8 ± 11.8
		N (%)
	**Sex**	
	female	6 (33.3%)
	male	12 (66.7%)

	**Corory heart disease**	
	without this disease	16 (88.9%)
	with this disease	2 (11.1%)
	**Hypertension**	
	without this disease	10 (55.6%)
	with this disease	8 (44.4%)
	**Diabetes**	
	without this disease	16 (88.9%)
	with this disease	2 (11.1%)
	**Cerebral infarction or cerebral hemorrhage**	
	without this disease	17 (94.4%)
	with this disease	1 (5.6%)

The above basic patient information and disease information was obtained from the patient's discharge medical records.
TAVI: post-transcatheter aortic valve implantation.

Table S2. The number of chest radiographs included in each label after reorganization. (A. MIMIC-CXR-JPG, CheXpert, NIH Chest X-ray Dataset, Montgomery County-X-ray Database, and Shenzhen Hospital CXR Set; B. COVID-19 RADIOGRAPHY, Chest X-Ray Images (Pneumonia), BRAX, VinDr-CXR, VinDr-PCXR, PLA General Hospital, and Fuwai Hospital dataset.)

Table S2 A

	MIMIC-CXR-JPG	CheXpert	NIH Chest X-ray Dataset	Montgomery County-X-ray Database	Shenzhen Hospital CXR Set
Atelectasis	65007	33456	11535	–	–
Cardiomegaly	64306	27068	2771	–	–
Consolidation	14635	14816	4667	–	–
COVID-19	–	–	–	–	–
Edema	36524	52291	2303	–	–
EffusionORPleuralEffusion	76917	86254	13307	–	–
Emphysema	–	–	2516	–	–
EnlargedCardiomediastinum	10002	10907	–	–	–
Fibrosis	–	–	1686	–	–
Fracture	7565	9040	–	–	–
Hernia	–	–	227	–	–
Infiltration	–	–	19870	–	–

LungLesion	10761	9187	–	–	–
LungOpacity	76375	105707	–	–	–
Mass	–	–	5746	–	–
Nodule	–	–	6323	–	–
NoFindingorNormal	143271	22419	60412	80	326
PleuralOther	3420	3524	3385	–	–
Pneumonia	26166	6047	1353	–	–
Pneumothorax	14217	19456	5298	–	–
SupportDevices	66558	116107	–	–	–
Tuberculosis	–	–	–	58	336
AorticEnlargement	–	–	–	–	–
Calcification	–	–	–	–	–
EnlargedPA	–	–	–	–	–
MediastinalShift	–	–	–	–	–
TAVI	–	–	–	–	–
Aortic dissection	–	–	–	–	–

Table S2 B

	COVID-19 RADIOGRAPHY	Chest X-Ray Images (Pneumonia)	BRAX	VinDr-CXR	VinDr-PCXR	PLA General Hospital	Fuwai Hospital
Atelectasis	–	–	3518	175	–	–	–
Cardiomegaly	–	–	3984	2119	–	–	–
Consolidation	–	–	3157	264	–	–	–
COVID-19	1200	–	–	–	–	–	–
Edema	–	–	50	5	–	–	–
EffusionORPleuralEffusion	–	–	1822	815	–	165	–
Emphysema	–	–	–	37	2	–	–
EnlargedCardiomediastinum	–	–	71	–	–	–	–
Fibrosis	–	–	–	1307	–	–	–
Fracture	–	–	624	73	–	–	–
Hernia	–	–	–	–	3	–	–
Infiltration	–	–	–	374	–	–	–
LungLesion	–	–	1290	–	–	–	–
LungOpacity	–	–	4065	766	–	313	–
Mass	–	–	–	–	–	–	–
Nodule	–	–	–	–	–	–	–
NoFindingorNormal	1341	1583	29009	12613	6050	165	5

PleuralOther	–	–	117	1255	–	–	–
Pneumonia	1345	–	774	769	487	–	–
Pneumothorax	–	–	214	84	–	118	–
SupportDevices	–	–	8791	–	–	–	–
Tuberculosis	–	4273	–	639	15	–	–
AorticEnlargement	–	–	–	2616	–	–	–
Calcification	–	–	–	425	–	–	–
EnlargedPA	–	–	–	164	–	–	–
MediastinalShift	–	–	–	110	–	–	–
TAVI	–	–	–	–	–	–	18
Aortic dissection	–	–	–	–	–	70	–

TAVI: post-transcatheter aortic valve implantation.
Many chest radiographs in the datasets were diagnosed with multiple labels, which resulted in a much larger number of chest radiographs in the reorganized datasets compared to the original ones.

Table S3. Model hyper-parameter details.

**Type**	**kernel_size**	**filters**	**strides**
**Input_block**			
**conv_block**	3	64, 64, 256	1,1
**identity_block**	3	64, 64, 256	2,2
**identity_block**	3	64, 64, 256	2,2
**conv_block**	3	128, 128, 512	2,2
**identity_block**	3	128, 128, 512	2,2
**identity_block**	3	128, 128, 512	2,2
**identity_block**	3	128, 128, 512	2,2
**conv_block**	3	128, 128, 512	2,2
**identity_block**	3	256, 256, 1024	2,2
**identity_block**	3	256, 256, 1024	2,2
**identity_block**	3	256, 256, 1024	2,2
**identity_block**	3	256, 256, 1024	2,2
**identity_block**	3	256, 256, 1024	2,2
**conv_block**	3	512, 512, 2048	2,2

**identity_block**	3	512, 512, 2048	2,2
**identity_block**	3	512, 512, 2048	2,2
**output_block**			

Table S4. Model training details.

optimizer	SGD
**momentum**	0.9
**lr_decay_type**	
**gpu**	1
**Init_lr**	0.01
**Min_lr**	0.0001
**Max Epoch**	100
**batch size**	16

Table S5. Summary of additional model evaluation metrics.

Metrics	F1-Score	Recall-Score	Top-1 Error	Top-2 Error	Top-4 Error	Top-6 Error	Top-8 Error	Specificity
**Value**	0.46	0.43	0.46	0.71	0.94	0.99	0.99	0.98

Table S6: Innovations and improvements of the model.

	Cardiomegaly	EffusionORPleuralEffusion	Pneumothorax	Edema
**PanCV_{soft}**	0.70	0.73	0.74	0.68
**PanCV**	0.73	0.75	0.81	0.77
**relative improvement**	3.84%	2.67%	9.36%	13.60%

Table S7. Summary of other related similar studies.

Authors	Year	Sample Size of Training Dataset	Abnormalities	Data resources	Method	Results	Ref.
**Eui Jin Hwang et al.**	2019	89,834 chest radiographs	Pulmonary malignant neoplasm, active tuberculosis, pneumonia, and pneumothorax	Single-center	Deep convolutional neural network with dense blocks and five parallel classifiers for	The algorithm demonstrated superior performance in analyzing chest radiographs when compared to physicians, including specialists in thoracic radiology,	[1]

					disease detection and localization	consistently surpassing them in accuracy	
**Ju Gang Nam et al.**	2021	43,292 chest radiographs	Malignant pulmonary nodules	Single-center	Deep convolutional neural network with 25 layers and eight residual connections, which trained in a semisupervised learning manner	The deep learning-based automatic detection algorithm surpassed physicians in the classification of malignant pulmonary nodules and detection performance on chest radiographs	2
**Fatemeh Homayounieh et al.**	2021	7,776 chest radiographs	Pulmonary lesions (including subtypes like mass, nodule, granuloma), pleural effusion, pneumothorax, atelectasis, and consolidation	Multi-center	Multi-scale structure capable of classifying and detecting multiple types of abnormalities in a single parametric learning model	Improved detection accuracy and AUC with AI assistance, especially beneficial for junior radiologists	3
**Ju Gang Nam et al.**	2021	146,717 chest radiographs	Pneumothorax, mediastinal widening, pneumoperitoneum, nodule/mass, consolidation, pleural effusion, linear atelectasis, fibrosis, calcification and cardiomegaly.	Single-center	Development of a ResNet34-based deep convolutional neural network for detecting and localizing major thoracic diseases	The algorithm significantly enhances the detection of 10 common abnormalities in chest radiographs, boosting radiologists' diagnostic accuracy and accelerating the reporting process for critical and urgent cases	4
**Jarrel C Y Seah et al.**	2021	821,681 chest radiographs	127 clinical findings	Multi-center	The deep-learning tool comprises three convolutional neural networks tailored for clinical decision support: an image projection classification model (attributes model), a clinical finding classification model (classification model), and a clinical finding segmentation model (segmentation model).	The model improves performance across most chest X-ray clinical findings	5
**Ngoc Huy**	2022	23,524	Aortic enlargement,	Multi-	The AI	The effectiveness of the	6

**Nguyen et al.**		chest radiographs	atelectasis, calcification, cardiomegaly, clavicle fracture, consolidation, emphysema, enlarged pulmonary artery, infiltration, interstitial lung disease, nodule/mass, opacity, pleural effusion, pleural thickening, pneumothorax, pulmonary fibrosis, rib fracture	center	computer-aided diagnosis system is directly integrated into the hospital's picture archiving and communication system after being trained on a fixed annotated dataset from other sources to investigate the performance of the system in real-world clinical settings and compare its effectiveness to the in-lab performance	AI system in identifying abnormal chest radiographs undergoes a significant decline when transitioning from training conditions to deployment in a clinical environment	
**Ju Sun et al.**	2022	95,363	COVID-19	Multi-center	The main model pipeline consists of lung segmentation, outlier detection, and feature extraction or classification modules.	AI-based tools have not yet reached their full diagnostic potential for COVID-19 and underperform compared to predictions made by radiologists	7
**Yaping Zhang et al.**	2023	5,091 chest radiographs	23 abnormal signs including synonyms, parasynonyms, or phrases that may appear in radiology reports	Multi-center	A bidirectional encoder representation from a transformers model is utilized to extract language entities and relationships from unstructured CXR reports, enabling the establishment of 23 labels for abnormal signs to train convolutional neural networks	The natural language processing (NLP)-generated captions of chest radiographs demonstrate robust consistency with those generated by expert radiologists, and the prior information provided by NLP is associated with enhanced efficiency in the reporting process	8

Reference

1. Hwang, E.J., Park, S., Jin, K.N., Kim, J.I., Choi, S.Y., Lee, J.H., Goo, J.M., Aum, J., Yim, J.J., Cohen, J.G., et al. (2019). Development and Validation of a Deep Learning-Based Automated Detection Algorithm for Major Thoracic Diseases on Chest Radiographs. JAMA Netw Open *2*, e191095. 10.1001/jamanetworkopen.2019.1095.

2. Nam, J.G., Park, S., Hwang, E.J., Lee, J.H., Jin, K.N., Lim, K.Y., Vu, T.H., Sohn, J.H., Hwang, S., Goo, J.M., and Park, C.M. (2019). Development and Validation of Deep Learning-based Automatic Detection Algorithm for Malignant Pulmonary Nodules on Chest Radiographs. Radiology *290*, 218-228. 10.1148/radiol.2018180237.

3. Homayounieh, F., Digumarthy, S., Ebrahimian, S., Rueckel, J., Hoppe, B.F., Sabel, B.O., Conjeti, S., Ridder, K., Sistermanns, M., Wang, L., et al. (2021). An Artificial Intelligence-Based Chest X-ray Model on Human Nodule Detection Accuracy From a Multicenter Study. JAMA Netw Open *4*, e2141096. 10.1001/jamanetworkopen.2021.41096.

4. Nam, J.G., Kim, M., Park, J., Hwang, E.J., Lee, J.H., Hong, J.H., Goo, J.M., and Park, C.M. (2021). Development and validation of a deep learning algorithm detecting 10 common abnormalities on chest radiographs. Eur Respir J *57*. 10.1183/13993003.03061-2020.

5. Seah, J.C.Y., Tang, C.H.M., Buchlak, Q.D., Holt, X.G., Wardman, J.B., Aimoldin, A., Esmaili, N., Ahmad, H., Pham, H., Lambert, J.F., et al. (2021). Effect of a comprehensive deep-learning model on the accuracy of chest x-ray interpretation by radiologists: a retrospective, multireader multicase study. Lancet Digit Health *3*, e496-e506. 10.1016/s2589-7500(21)00106-0.

6. Nguyen, N.H., Nguyen, H.Q., Nguyen, N.T., Nguyen, T.V., Pham, H.H., and Nguyen, T.N. (2022). Deployment and validation of an AI system for detecting abnormal chest radiographs in clinical settings. Front Digit Health *4*, 890759. 10.3389/fdgth.2022.890759.

7. Sun, J., Peng, L., Li, T., Adila, D., Zaiman, Z., Melton-Meaux, G.B., Ingraham, N.E., Murray, E., Boley, D., Switzer, S., et al. (2022). Performance of a Chest Radiograph AI Diagnostic Tool for COVID-19: A Prospective Observational Study. Radiol Artif Intell *4*, e210217. 10.1148/ryai.210217.

8. Zhang, Y., Liu, M., Zhang, L., Wang, L., Zhao, K., Hu, S., Chen, X., and Xie, X. (2023). Comparison of Chest Radiograph Captions Based on Natural Language Processing vs Completed by Radiologists. JAMA Netw Open *6*, e2255113. 10.1001/jamanetworkopen.2022.55113.
